# Enhancement of Per- and Polyfluoroalkyl Substance Uptake from Contaminated Soil by Applying Plant Growth Regulators: Screening and Cross-Species Evaluation

**DOI:** 10.1021/acsestengg.6c00068

**Published:** 2026-06-05

**Authors:** Aswin Kumar Ilango, Oya Aydin Urucu, Madhav Kharel, Yanna Liang

**Affiliations:** a Department of Environmental and Sustainable Engineering, University at Albany, State University of New York, Albany, New York 12222, United States; b Department of Chemistry, Marmara University Faculty of Sciences, Istanbul 34722, Turkey

**Keywords:** PFAS, effect of PGRs, phytoaccumulation, Timothy grass, alfalfa

## Abstract

Innovative approaches are urgently needed to improve the phytoremediation of complex mixtures of per- and polyfluoroalkyl substances (PFAS) in contaminated soils. This study investigated the effectiveness of plant growth regulators (PGRs) in enhancing the uptake of a mixture of eight PFAS (100 μg/kg each) by Timothy grass (TG) from soil. A suite of PGRs including auxins (indole-3-acetic acid [IAA], indole-3-butyric acid [IBA]), gibberellic acid (GA), jasmonic acid, α-naphthaleneacetic acid, and 2,4-dichlorophenoxyacetic acid) was applied individually via foliar spray at concentrations ranging from 0.1 to 200 μM. Among all treatments, IBA at 10 μM (IBA-10), IAA-1, and GA at 0.1–1 μM significantly enhanced shoot accumulation of PFHxA and PFHpA, with maximum uptake reaching ∼23% in TG during the second harvest (days 55–108). Compared to the controls without exposure to PGRs, this enhancement was ∼63%. In addition, PGRs enhanced the translocation of long-chain PFOS and PFNA, with increases of 45–82% over the controls. Uptake of ΣPFAS peaked around 7–9% of the total spiked mass for those with IBA-10, IAA-1, or GA-1/GB-0.1, underscoring the importance of type and concentration of PGRs in enhancing phytoextraction. Similar results were also observed for alfalfa undergoing similar treatment with PGRs. Insight from both TG and alfalfa indicates that not all PGR applications led to enhanced biomass weight and PFAS uptake percentage. Some treatments increased tissue PFAS concentrations without improving biomass dry weight, while others enhanced biomass but resulted in only modest increases in PFAS translocation. These results demonstrate that some foliar-applied phytohormones can regulate PFAS accumulation in plants, offering a potential strategy to improve phytoextraction efficiency. The underlying mechanisms, however, will need to be further elucidated.

## Graphical abstract

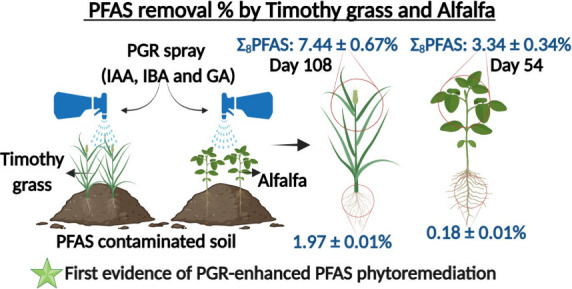

## 1 Introduction

PFAS are a class of synthetic compounds characterized by strong carbon–fluorine bonds that confer extreme chemical stability and resistance to degradation.^1–3^ Since the 1940s, perfluoroalkyl acids (PFAAs) including perfluorooctanoic acid (PFOA) and perfluorooctanesulfonic acid (PFOS) have been extensively used in industrial and consumer products such as aqueous film-forming foams (AFFF), nonstick coatings, textiles, and food packaging.^4,5^ Their persistence and mobility have led to widespread environmental contamination, with PFAS now detected in groundwater, soils, crops, and wildlife worldwide.^6,7^ The chronic PFAS exposure has been linked to hepatotoxicity, endocrine disruption, and developmental abnormalities.^8,9^

While significant advances have been made in treating PFAS-contaminated water using adsorptive^10–14^ or destructive technologies,^15–18^ removing PFAS from soils remains particularly challenging. The soil matrices offer complex interactions between PFAS and organic matter and clay particles, which may either increase or decrease the mobility and phyto/bioavailability of PFAS, reducing the efficacy of conventional remediation approaches, such as soil washing, pump-and-treat, in situ flushing, and bioremediation.^19–23^ This is especially concerning in agricultural systems where biosolids, wastewater irrigation, and atmospheric deposition contribute to PFAS accumulation,^24–26^ threatening food safety and long-term soil health.

The environmental concentrations of PFAS vary widely depending on sources and the presence of unknown precursor compounds that can transform into stable PFAAs. For example, biosolids from wastewater treatment plants (WWTPs) in the United States contained PFOA, PFOS, and PFDA at concentrations ranging from 20 to 400 μg/kg dry weight.^27^ In our previous study, biosolids from WWTPs in Maine, USA contained total PFAS concentrations of approximately 105 μg/kg.^28^ In addition to legacy PFAS, PFAS precursors and emerging alternatives are also commonly detected in biosolids. These precursors can transform into stable PFAAs over time under suitable environmental conditions.

Phytoremediation is a cost-effective and environmentally sustainable strategy that utilizes plants to extract, stabilize, or degrade environmental contaminants, including PFAS, from contaminated soils.^29–31^ Soil amendments such as biochar have shown promise in influencing PFAS mobility and phytoavailability.^32–34^ In our recent study, the application of a low biochar dose (0.05% w/w) enhanced the uptake of PFOA by approximately 10% in shoots of Conservation Reserve Program (CRP)’s seed mix cultivated in Scantic soil mixed with biosolids.^28^ On the other hand, a higher biochar amendment (1% w/w) effectively immobilized PFAS in the soil matrix, thereby limiting their translocation to aboveground plant tissues. Mobilization by adding the anionic surfactant (i.e., sodium dodecyl sulfate, SDS)^34^ to biosolids-amended soil at a dose range of 10–100 mg/kg significantly increased the plant uptake of ΣPFAS by 15.48%–108.57% compared to the controls. Although lower-molecular-weight PFAS including short chain and GenX tend to accumulate in the harvestable shoots of grasses and legumes, the uptake of long-chain PFAS has been a significant challenge in the field of phytoremediation of these contaminants.^35–38^ Furthermore, while soil amendments may increase PFAS phytoavailability, the amendments can also alter soil chemistry and potentially affect soil health,^39,40^ rendering soil undesirable for growing crops. Therefore, innovative approaches for improving PFAS uptake efficiency are essential for advancing phytoremediation as a viable strategy for large-scale soil remediation, while avoiding unintended alterations to bulk soil physicochemical properties.

Plant growth regulators (PGRs) are naturally occurring phytohormones or synthetic analogues widely employed in agriculture, horticulture, and viticulture to modulate plant growth and development.^41,42^ They regulate key physiological processes including cell division, elongation, differentiation, and responses to environmental stress. In addition to promoting overall plant vigor, PGRs have been shown to enhance seed germination, stimulate root and shoot development, improve nutrient uptake efficiency, and increase plant tolerance to both abiotic and biotic stressors.^43,44^ Key classes of PGRs such as auxins (e.g., indole-3-acetic acid [IAA] and indole-3-butyric acid [IBA]), gibberellic acid (GA), jasmonic acid (JA), α-naphthaleneacetic acid (NAA), and 2,4-dichlorophenoxyacetic acid (DCPAA) are reported to alter root architecture, enhance membrane permeability, and regulate xylem loading in the plant tissues.^43,44^

Specifically, auxins (IAA and IBA) promote cell division and elongation, stimulate root development, and enhance overall biomass production.^45,46^ The increased root surface area and shoot biomass may enhance PFAS uptake and total contaminant removal when plant–soil partitioning remains constant. GA, a diterpenoid phytohormone, promotes stem elongation, delays dormancy in grasses, and enhances nutrient uptake and membrane permeability. GA also improves tolerance to abiotic stresses by regulating antioxidant metabolism,^47,48^ potentially extending the effective growing season and increasing cumulative PFAS removal. JA functions as a signaling molecule that activates plant defense pathways and enhances tolerance to abiotic stress, which may help sustain growth under contaminant-induced stress conditions.^49,50^ Similarly, both NAA and DCPAA would stimulate cell division and elongation, contributing to enhanced biomass production.^51,52^ In parallel, a substantial body of literature demonstrates that PGRs influence contaminant dynamics in plants exposed to heavy metals such as Cd, Pb, and Zn in contaminated soil. Hormone-mediated modulation of root morphology, antioxidant systems, and membrane-transport processes has been shown to affect metal uptake, translocation, and stress tolerance in a species- and dose-dependent manner.^53–55^ Overall, these PGRs were selected because their positive effects on biomass production, root architecture, membrane transport, and stress tolerance are mechanistically linked to improved phytoremediation performance. These physiological changes have direct implications for the uptake and internal transport of contaminants. However, while the role of PGRs in improving plant growth under environmental stress is well documented, their potential to enhance the uptake and translocation of persistent organic pollutants such as PFAS has not been experimentally investigated.

The objective of this study was to systematically evaluate the effects of foliar-applied PGRs on the uptake of eight PFAS by Timothy grass (TG) from contaminated soil. We selected a spiking concentration of 100 μg/kg for each of the eight PFAS, as this concentration is environmentally relevant and enables reliable quantification of PFAS uptake under controlled conditions. Six PGRs were chosen based on their documented physiological functions and applied at varying concentrations (0.1–200 μM) during two distinct growth stages. We hypothesized that (1) foliar application of specific PGRs could significantly enhance PFAS uptake and root-to-shoot translocation in TG in a dose-dependent manner and (2) if PFAS partitioning in soil remains the same, increased biomass upon foliar PGR application can lead to greater total PFAS removal. Once the PGRs’ effect on TG’s translocation of PFAS was known, a follow-up experiment using alfalfa was conducted to assess cross-species reproducibility of the PGR’s effect. This study presents the first experimental evidence of hormone-assisted PFAS phytoextraction, offering novel insights into practical strategies for enhancing PFAS removal from soil. At the same time, issues and challenges relevant to this approach were discussed as well.

## 2 Materials and Methods

### 2.1 PFAS Soil, Cultivation, PGR Treatments, and Plant Harvesting

#### 2.1.1 Preparation of PFAS Soil Mixture for Plant Growth

To mimic realistic contamination, a PFAS-free local soil collected from Albany, NY. This soil is classified as sandy loam, with a pH of 7.38 ± 0.41, content of soil organic matter of 4.82 ± 0.12%, and total organic carbon of 2.44 ± 0.45%.^56^ Background PFAS concentrations were analyzed according to USEPA Method 1633 with minor modifications.^57^ Only trace levels of PFOA and PFNA were detected across all soil replicates (Table S1), and the soil was therefore considered PFAS-free for subsequent experiments. The local soil was then spiked with eight PFAS, namely, PFHxA, PFHpA, PFOA, PFNA, PFBS, PFHxS, PFOS, and GenX at 100 μg/kg each. The PFAS spiked soils were kept for aging for 3 months under laboratory conditions to ensure equilibration and uniform PFAS distribution. To assess the stability of the spiked PFAS, the soils were measured after an aging period and no significant variation in PFAS concentrations was observed, confirming the stability of the spiked PFAS in soil (Table S1).

The spiked soil was homogenized and distributed into polyethylene containers (1 kg soil per container) for subsequent cultivation experiments. Approximately 1.5 g of TG seeds were sown on November 13, 2024, into PFAS-contaminated soil. Following seed germination, the pots were transferred to a controlled greenhouse at the University at Albany, where temperature (20–25 °C), relative humidity (40–60%), and the soil moisture were maintained by daily watering to near field capacity (80%).^34^ The nutrient supply was provided using 20% strength Hoagland solution as the source of N, P, and K. In addition, the natural daylight supplemented with artificial lighting (10 h/day) as needed with an intensity of ∼200–300 μmol m^–2^ s^–1^.

#### 2.1.2 Foliar Applications of PGRs

To examine the effect of exogenous phytohormones, six different PGRs including IAA, IBA, GA, JA, DCPAA, and NAA were separately applied via foliar spraying at concentrations of 1, 10, or 100 μM. The PGR concentrations used in this study (1–100 μM) correspond to approximately 0.2–35 mg/L depending on each PGR’s molecular weight. Foliar applications of auxins and gibberellins in agricultural practice are commonly conducted within low-to-moderate mg/L solution ranges, frequently reported between approximately 1 and 200 mg/L depending on crop species and growth stages.^58,59,41^ The spraying of different volume of each freshly prepared PGR solution took place on days 27, 34, 41, and 48 (Table S2). During days 27 and 34, when plant biomass was relatively low, 2.5 mL of PGR solution per pot at the designated concentrations was applied. As plant biomass increased, the application volume was increased to 5 mL per pot on days 41 and 48. Following the first harvest, to evaluate the effects of higher application volumes of PGR, 25 mL per pot of selected PGR solutions was applied on days 91, 98, and 105.

PGRs were applied individually to each pot using a handheld sprayer in a separate area to prevent cross-contamination. The solutions were applied as a uniform foliar spray directly onto the leaf surfaces until the leaves were evenly wetted without visible runoff or dripping. Control plants were sprayed with equivalent volumes of deionized water by using the identical spraying procedure. Foliar delivery allows PGRs to enter systemic circulation via the phloem to regulate belowground processes, including root initiation, elongation, and hormonal cross-talk that influence nutrient and xenobiotic translocation. This avoids confounding effects associated with soil-drench treatments (e.g., altered PFAS sorption/partitioning in soil, competition with dissolved organics, and changes to soil PFAS mobility) that could obscure mechanistic interpretations related to plant physiology. The first shoot harvest using acid-washed stainless-steel tools was conducted on day 54 after seeding, followed by rinsing with deionized water, weighing the wet biomass, biomass freeze-drying, and PFAS extraction detailed in the following.

After the first cut, based on the lack of increase of PFAS mass uptaken by TG shoots in comparison to the controls, JA, NAA, and DCPAA were excluded from further studies. The pots that received these three PGRs were sprayed with the remaining PGRs, such as IAA at 0.5, 1, 10, 100, or 200 μM; IBA at 5, 10, 15, 20, or 50 μM; and GA at 0.1, 0.2, 0.5, 1, or 5 μM. For pots receiving the same PGR at the same dose, for example, IAA at 1 μM, it was labeled as IAA-1-C with C standing for continuous application. For pots that were sprayed with different PGRs, such as JA at 1 μM during the first 54 days and then IAA at 0.5 μM during the second phase, it was labeled as JA-1/IAA-0.5.

During the second phase of growth in the same greenhouse under identical conditions, the selected PGRs at designated doses were sprayed to TG on days 91, 98, and 105 as detailed in Table S2. The second harvest took place on day 108, with shoot and root biomass collected separately for PFAS extraction and analysis. Once the total uptake of PFAS by TG shoots under different conditions was calculated, the three PGRs with different doses were downselected again. TG roots of those leading to a significant increase of overall shoot accumulation of individual and total PFAS were weighed and extracted for PFAS. The best treatment conditions then took both the shoot and root uptake into account.

To assess whether the best PGR treatments could also lead to enhanced PFAS uptake in other plants, alfalfa was used as an evaluation species. Approximately, 0.5 g of alfalfa seeds was evenly sown in each pot containing 250 g of soil. The germinated seedlings were watered daily to maintain soil moisture at 80% field capacity by keeping the container weight constant.^34^ The same four-weekly foliar spray schedule was followed (days 27, 34, 41, and 48), using the three most effective PGRs, IAA, IBA, and GA at different concentrations (Table S3). Although alfalfa plants appeared smaller in height, their broader leaves and more branched canopy required adequate foliar coverage. Thus, the same spray volume used for TG during the second harvest was maintained for alfalfa to ensure consistent foliar exposure across treatments. Alfalfa shoots and roots were harvested on day 54, dried, and processed identically to TG for both biomass evaluation and PFAS extraction.

### 2.2 PFAS Extraction from Complex Matrices

#### 2.2.1 Extraction of PFAS from the Spiked Soil

PFAS concentrations in the aged soils were quantified at zero in time using USEPA Method 1633 with slight modifications.^57,28,60^ Briefly, 5 g (dry wt.) of soil from each replicate was spiked with 20 ng of both perfluoro-*n*-[1,2,3,4,6-^13^C_5_]­hexanoic acid (^13^C_5_-PFHxA) and perfluoro-*n*-[1,2,3,4,5-^13^C_5_]­hexane-1-sulfonic acid (^13^C_5_-PFHxS) as an extracted internal standard (EIS). The samples were then vortexed and equilibrated for 30 min. Subsequently, 10 mL of 0.3% methanolic NH_4_OH was added to each sample, followed by vortexing and mixing on a variable shaker. After 30 min, samples were centrifuged at 4500 rpm for 10 min, and the supernatant was transferred to a new tube. The extraction procedure was repeated twice by adding 5 and 15 mL of methanolic ammonium hydroxide to the residual soil, and the resulting supernatants were combined in the same collection tube. The combined extracts were treated with powdered Envi-Carb carbon for matrix cleanup, concentrated under a N_2_ evaporator, and pH-adjusted to 6.5 ± 0.5 using HCOOH and NH_4_OH. Solid-phase extraction was performed using Bond Elut WAX cartridges, and PFAS were eluted with 1% methanolic NH_4_OH, followed by the addition of 25 μL of CH_3_COOH. Final extracts were quantified by LC-MS/MS.

#### 2.2.2 Extraction of PFAS from TG and Alfalfa

To extract PFAS from both TG and Alfalfa, shoots and roots from both plants were freeze-dried at −55 °C for 48 h followed by homogenization using a blender. The powdered biomass samples were then subjected to extraction using our previously reported MTBE-NaOH method, with minor modifications.^28,34,61^ Briefly, 0.2–0.5 g of dry biomass was placed in 50 mL clean polypropylene tubes. Quality control (QC) samples, including method blanks using Ottawa sand; low-level ongoing precision and recovery standards (LLOPR), and midlevel OPR (MLOPR) using the biomass from the controls without exposure to PFAS were set up as well.

Before extraction, each biomass sample was spiked with 20 ng of ^13^C_5_-PFHxA and ^13^C_5_-PFHxS as an extracted internal standard (EIS), followed by the addition of 4 mL of 0.4 M NaOH. The samples were then left at 4 °C overnight to enhance the breakdown of the plant cell walls and membranes. The sample was then treated with 2 mL of 0.5 M tetrabutylammonium hydrogen sulfate and 4 mL of 0.25 M Na_2_CO_3_ buffer, followed by the addition of 5 mL of MTBE. After thorough mixing, the mixture was shaken for 30 min at 120 rotation per minute (rpm) and then centrifuged [4500 rpm with a relative centrifugal force (RCF) of ∼1920*g*, 15 min] to separate the organic and aqueous layers. The MTBE layer containing PFAS was collected in a new PP tube, and the remaining aqueous and solid slurry was re-extracted twice with MTBE (5 mL) to ensure complete PFAS extraction. The combined organic extracts (∼13–14 mL) were then evaporated under N_2_, and the residue was reconstituted in water containing <20% methanol. The solution was then pH-balanced (pH 6.5–7 ± 0.5) and loaded onto an Agilent Bond Elut WAX cartridge for cleanup. Finally, the PFAS retained on the cartridges were eluted with 5 mL of 1% basified methanol. The chemical reagents used in this study are detailed in Table S4, and the physiochemical properties of PFAS examined in this study are shown in Table S5. The extraction efficiency (%) of individual and ΣPFAS compounds in soil and PFAS concentrations (ng/g) and removal efficiencies (%) of individual and ΣPFAS compounds in plant shoots and roots (ng/g dry weight) were calculated using eqs S1–S3, as described in Supporting Information Text S1.

### 2.3 PFAS Quantifications

PFAS analysis was performed using an Agilent 6470 Triple Quadrupole LC/MS/MS, as described in our previous reports and in Text S2 and Table S6.^11,34,62,11^ For all analysis of sample batches, matrix, instrumental, and method blanks were included to assess potential contamination from different sources, such as reagents used, instrument, etc. The MDLs and LOQ were defined as the concentrations with signal-to-noise ratios of 3 and 10, respectively. The PFAS with concentrations below MDLs were reported as nondetected (ND). The EIS recoveries for target PFAS compounds typically ranged from 70% to 115%, consistent with the method performance criteria established in EPA Method 1633.^57^ The corresponding QC results for the target PFAS in the biomass matrix are presented in Table S7.

### 2.4 Data Analysis

Data are reported as mean ± standard deviation (SD) (*n* = 3). Statistical analysis was performed using one-way analysis of variance (ANOVA) in R statistical software to compare treatments with the control. When significant differences were observed, Tukey’s Honestly Significant Difference (HSD) post hoc test was performed for multiple comparisons. Statistical significance was defined as *p* < 0.05.

## 3 Results and Discussion

### 3.1 Initial Screening of the Six PGRs through Foliar Application to TG during the First 54 Days

#### 3.1.1 Effects of PGRs on TG Growth in 54 Days

Upon the first harvest on day 54, TG shoot biomass varied significantly across PGR treatments, as shown in Table S8. Among these, IBA at 10 μM (IBA-10) produced the highest shoot biomass of 3.40 ± 0.27 g, notably surpassing the 2.04 ± 0.03 g from the PFAS-contaminated control without any PGRs (control-2). In contrast, other PGRs such as GA, JA, DCPAA, and NAA failed to enhance growth, with shoot biomass remaining below the control-2 level across all tested concentrations (1–100 μM). The TG biomass from control-1 (no PFAS, no PGRs) was 2.01 ± 0.04 g, which was similar to that of control-2 (PFAS, no PGRs) (2.04 ± 0.03 g). Thus, the PFAS at the studied concentration did not appear to impact TG growth negatively.

#### 3.1.2 Individual PFAS Uptake (%) in TG Shoots by Day 54

Uptake of PFAS is expressed as the total mass of PFAS accumulated in plant tissues, reported as percent removal from the soil system, rather than as concentration- or phytoaccumulation-based metrics. The uptake of individual PFAS varied substantially across treatments, with short-chain compounds exhibiting greater mobility and enhanced responsiveness to PGR application in TG shoots at day 54, as illustrated in Figure 1. One-way ANOVA showed that IBA-10 led to statistically significant higher uptake of PFHxA, PFHpA, and PFBS in comparison with the controls. The maximum PFHxA uptake (7.14 ± 0.74%) was 89.4% increase compared to the control-2 (3.77 ± 0.23%), followed by significant uptake of PFHpA (1.59 ± 0.09%) and PFBS (2.78 ± 0.58%) with this condition. For PFHpA and PFBS, the increase in comparison with the control-2 (0.82 ± 0.04% and 1.27 ± 0.19%) was 93.9% and 119%, respectively. These compounds are known to exhibit higher aqueous solubility and lower soil sorption affinity, making them more readily translocated via xylem to aerial tissues.^63,64^ PGR-induced enhancement likely results from hormone-mediated stimulation of root, xylem, and transpiration rates, which could facilitate mass flow of mobile contaminants.

**Figure 1:**
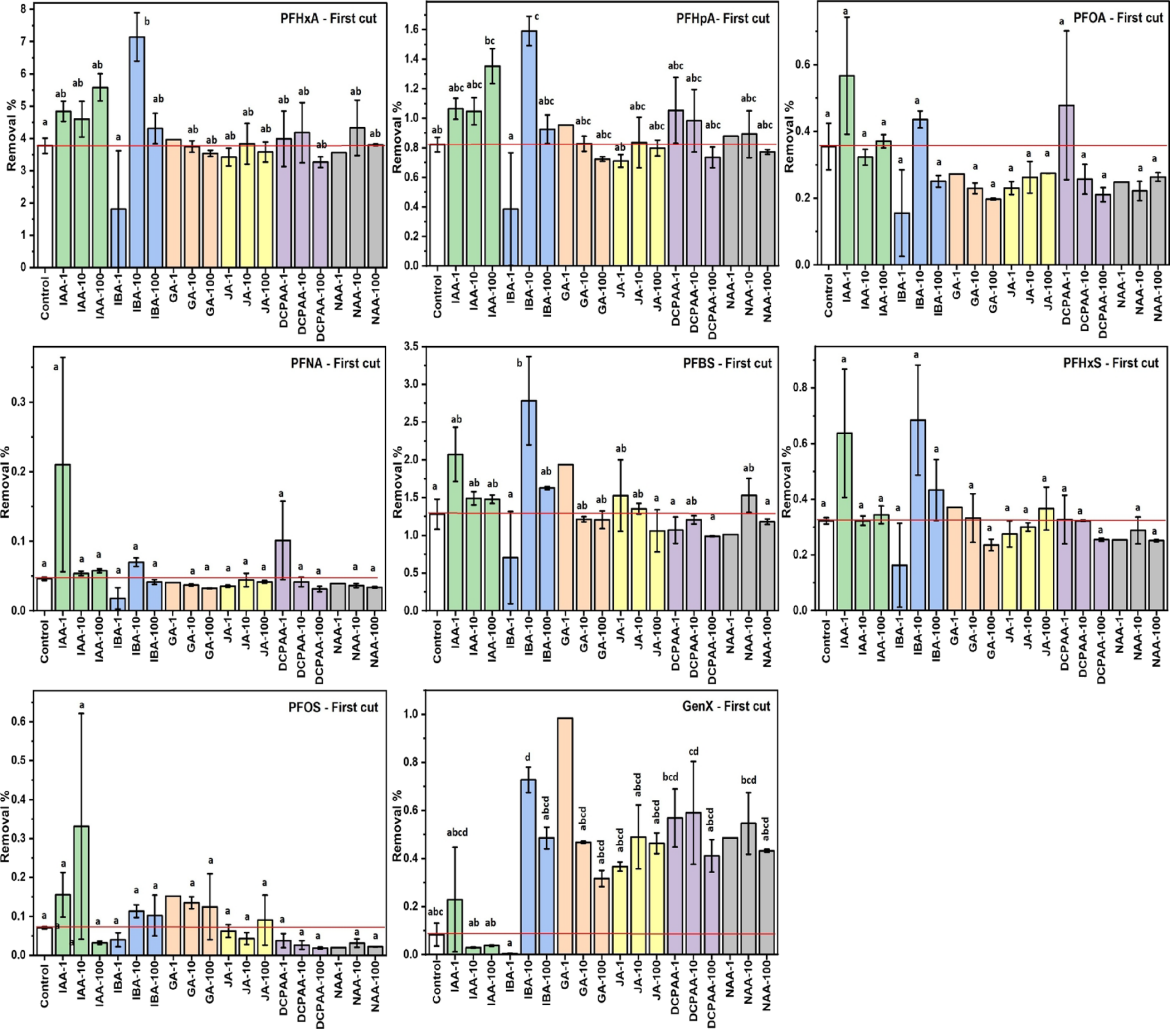
Removal (%) of individual PFAS from soil by TG during the first harvest (day 54) under foliar application of various PGRs. Red horizontal lines indicate average removal in the control (control-2) without exposure to PGRs (*n* = 3). Statistical significance was evaluated using one-way ANOVA followed by Tukey’s Honestly Significant Difference (HSD) test. Letters represent significant differences among the treatments with TG shoots (*p* < 0.05).

One-way ANOVA revealed that long-chain PFAS, such as PFOA, PFNA, and PFOS, did not show significant differences in TG shoots during the first cut (day 54) with PGR treatments (Figure 1). For these PFAS, limited shoot accumulation (<0.7%) was observed across all treatments, reflecting their stronger sorption to soil and roots and limited xylem mobility due to higher hydrophobicity.^65,66^ Given marginal uptake enhancements with the studied PGRs, alternative strategies for translocating long-chain PFAS effectively to the harvestable TG shoots are urgently needed. With respect to GenX, all PGRs at all tested doses, except IAA, increased the uptake of this PFAS by the TG shoots. The highest accumulation (0.98%) occurred with GA-1, approximately 5-fold higher than the control-2 (0.22 ± 0.21%), followed by IBA-10, JA-10, DCPAA-10, and NAA-10. The tested PGRs increased the concentrations of individual PFAS and Σ_8_PFAS (ng/g) in TG shoots on day 54, as discussed in Text S3 and Figure S1.

### 3.2 Screening of Downselected PGR Treatments Applied to TG between Days 55 and 108

#### 3.2.1 TG Growth during Days 55 and 108

As detailed above, due to lack of enhancement of PFAS uptake for those exposed to JA, NAA, or DCPAA by TG shoots on day 54, these three PGRs were excluded from further evaluation. All pots were continued in the greenhouse although some of them received different PGRs than those during the first 54 days (Table S2). It is known that the effect of PGRs on plant growth is dose-dependent. For example, moderate auxin concentrations (5.7–57 μM) enhance shoot and root biomass, chlorophyll content, and overall plant vigor, whereas higher doses (>570 μM) often inhibit growth due to auxin-induced toxicity or oxidative stress responses.^67^ Thus, to understand the dose effect on TG uptake of PFAS better, during the second phase growth from day 54 to day 108, the doses of the downselected PGRs, IAA, IBA and GA were expanded as shown in Table S3.

As shown in Table S9, foliar application of IBA-10-C as well as the combinations NAA-1/IBA-10a, NAA-10/IBA-10b, and NAA-100/IBA-10c consistently yielded the highest TG shoot biomass (2.73 ± 0.06 to 3.49 ± 0.10 g), significantly exceeding the control-2 (2.46 ± 0.20 g). However, increasing the IBA concentration beyond 10 μM (e.g., IBA-100/IBA-15, JA-100/IBA-20, and DCPAA-1/IBA-50) did not result in further biomass enhancement, suggesting a possible saturation threshold or potential growth inhibition at higher doses. In addition, IAA at all doses, 0.5–200 μM (JA-1/IAA-0.5, IAA-1-C, IAA-10-C, IAA-100-C, and JA-10/IAA-200) failed to improve biomass relative to the control-2. In contrast, GA demonstrated a clear dose response showing a bell curve with GA-100/GA-0.5 leading to a dry biomass of 3.16 ± 0.02 g. Over the entire growth period (day 1 to day 108), total biomass was very similar between control-1 (4.53 ± 0.16 g) and control-2 (4.50 ± 0.23 g) (sum of respective values from Tables S8 and S9). This again indicates that PFAS exposure did not negatively affect plant growth under the experimental conditions.

#### 3.2.2 Individual PFAS Uptake (%) in TG Shoots between Days 55 and 108

Figure 2 shows PFAS removal % from soil by TG shoots at the second harvest (day 108) under chosen PGR treatments. The main observation is that TG shoot accumulation of short-chain PFAS such as PFHxA, PFHpA, and PFBS showed clear concentration-dependent responses to specific PGRs. For PFHxA, several treatments significantly outperformed the control, with IAA-1-C, GA-1/GA-0.1, GA-10/GA-0.2, GA-100/GA-0.5, and DCPAA-100/GA-5 yielding uptake values exceeding 20% and in some cases approaching 30%. Notably, one-way ANOVA showed that GA showed similar uptake in both lower (GA-1/GA-0.1) and higher concentration (GA-100/GA-0.5), indicating an optimal concentration range for uptake enhancement i.e., GA-1/GA-0.1. For PFHpA, the most positive treatments were IAA-1-C, IBA-10-C, GA-1/GA-0.1, and DCPAA-100/GA-5, which achieved uptake ranging from 7.77 ± 0.65% to 9.97 ± 0.003%, well above the control-2 (5.67 ± 0.36%).

**Figure 2:**
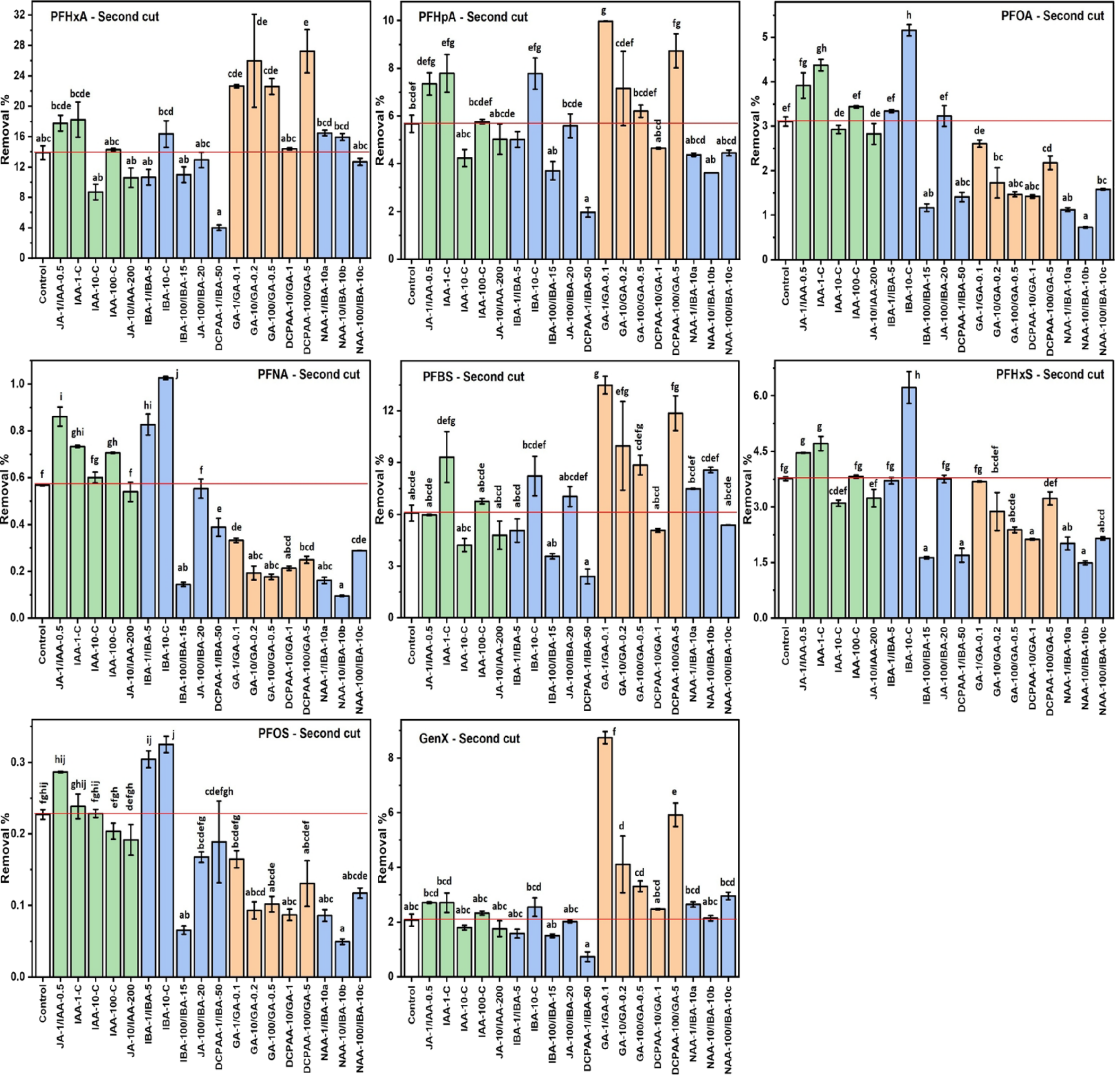
Removal (%) of individual PFAS from soil by TG shoots during the second harvest (days 55–108) following the chosen foliar application of PGRs. Red horizontal lines indicate average removal in the control (control-2) without exposure to PGRs (*n* = 3). Statistical significance was evaluated using one-way ANOVA followed by Tukey’s Honestly Significant Difference (HSD) test. Letters represent significant differences among the treatments with TG shoot (*p* < 0.05).

According to one-way ANOVA, PFBS uptake was significantly enhanced under GA-1/GA-0.1 (13.5 ± 0.51%) compared to control-2 (6.08 ± 0.48%), supporting the idea that low-dose GA can enhance translocation of short-chain PFAS. These compounds benefit from higher solubility and reduced hydrophobicity, facilitating xylem mobility under hormone-optimized conditions.^68,69^ Although PFHxS exhibited generally lower accumulation compared to the shorter chain PFAS, improvements were observed under IBA-10-C, with uptake of 6.22 ± 0.42%, higher than the control-2 (3.76 ± 0.05%).

Similarly, for PFOS, IAA-1-C and IBA-10-C led to slight, but not statistically significant increase in uptake of 0.23 ± 0.01% and 0.32 ± 0.01%, respectively, relative to the control-2 (0.22 ± 0.01%). Overall, this indicates a limited but measurable response of long-chain PFAS to auxin treatments. For both PFOA and PFNA, foliar application of GA at all tested concentrations (0.1–5 μM) did not enhance TG shoot accumulation, indicating limited effectiveness of gibberellins for long-chain PFCAs (Figure 2). In contrast, auxin-based treatments, particularly IAA at 1 μM and IBA at 10 μM, significantly improved the accumulation of these compounds. Regarding GenX, notably, GA-1/GA-0.1 was the only treatment that markedly enhanced the uptake of this PFOA replacement, achieving 8.74 ± 0.22% accumulation in harvestable TG shoots, which was 3-fold greater than the control-2 (2.07 ± 0.22%).

The observed enhancement PFAS uptake by GA treatments (0.1–5 μM) aligns with existing literature demonstrating a biphasic, dose-dependent effect of gibberellins on plant development. For example, Castro-Camba et al. (2022) reported that foliar application of GA_3_ at low micromolar concentrations (1–5 μM) significantly stimulated chlorophyll content, stem elongation, and shoot biomass in tomato seedlings, whereas higher concentrations failed to produce additional benefits, suggesting a saturation threshold or negative feedback on growth-related signaling pathways.^70^ This supports our observations that low-dose GA application enhanced shoot development and PFAS uptake % while avoiding the growth-inhibitory effects associated with higher concentrations. Overall, these results emphasize that auxin-based PGRs (particularly IBA and IAA at intermediate concentrations) and GA at low concentration remain as the most effective in enhancing PFAS accumulation in TG shoots. The tested PGRs increased the concentrations of individual PFAS and Σ_8_PFAS (ng/g) in TG shoots on day 108, as discussed in Text S4 and Figure S2.

### 3.3 Total PFAS Uptake (%) by Two Cuts of TG Shoots

The total PFAS removal from soil, measured exclusively through TG shoot accumulation, showed marked differences across PGR treatments and between the two harvests (Figure 3). For the first cut (day 54), one-way ANOVA confirmed that only IBA-10 resulted in the statistically significant higher uptake (1.69 ± 0.21%), nearly doubling the removal compared to the control-2 (0.84 ± 0.02%). IAA-1, IAA-100, and GA-1 had modest improvements over control-2 levels, but these differences were not statistically significant. In comparison, the growth between day 54 and day 108 exhibited a substantial increase in Σ_8_PFAS uptake across several PGR conditions. According to one-way ANOVA, IAA-1-C and IBA-10-C, as well as GA-1/GA-0.1 and DCPAA-100/GA-5, demonstrated the highest PFAS removal, ranging from 6.01 ± 0.65% to 7.44 ± 0.67% compared to control-2 (4.42 ± 0.26%).

**Figure 3:**
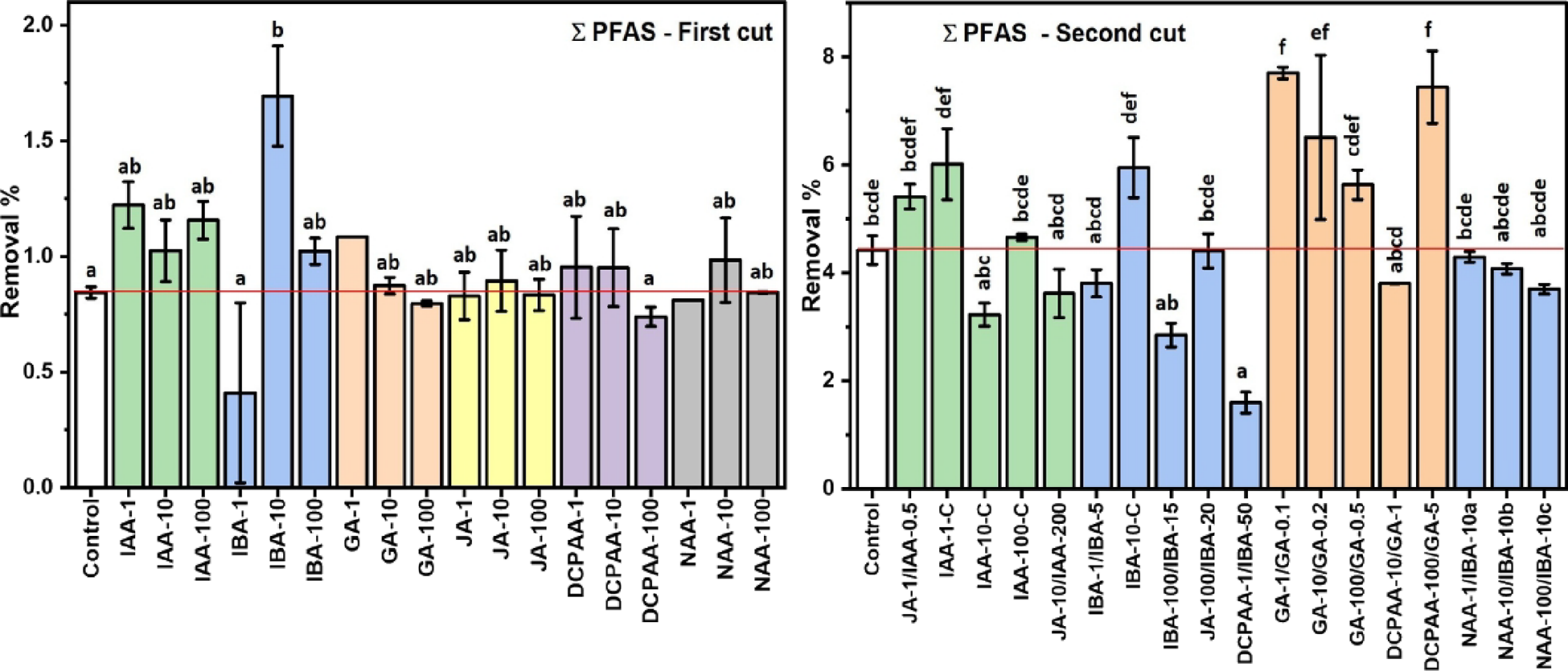
Total PFAS removal (%) from soil by TG shoots during the first (day 54) and second (day 108) harvests under various foliar applications of PGRs. Treatments include IAA, IBA, GA, JA, DCPAA, and NAA at different concentrations. Red horizontal lines indicate average removal in the control (control-2) without exposure to PGRs (*n* = 3). Statistical significance was evaluated using one-way ANOVA followed by Tukey’s Honestly Significant Difference (HSD) test. Letters represent significant differences among the treatments with TG shoots (*p* < 0.05).

The increased uptake of PFAS by the second cut was in line with our previous observations^34^ where sodium dodecyl sulfate (SDS) was applied to biosolids-amended soils (spiked with an 8-PFAS mixture, 300 μg/kg each) as a mobilizing agent to increase PFAS availability to TG. The SDS treatment led to higher uptake of Σ_8_PFAS by the second cut (days 40 and 80) than the first cut. This could be due to more established root systems facilitating faster and more PFAS accumulation during the later growth stage of TG.

It is also important to note that the Σ_8_PFAS uptake % from all pots treated with IBA-10 (a–c), which were previously exposed to NAA (100, 10, or 1 μM), was lower (3.69 ± 0.08% to 4.29 ± 0.09%) than the control-2 (4.42 ± 0.26%) without exposure to any PGRs. The reduced PFAS removal % in IBA-10-treated TG plants previously exposed to NAA may suggest potential antagonistic hormonal interactions,^71^ where early application has altered auxin signaling or reduced tissue responsiveness to subsequent IBA application. Such interactions highlight the complexity of hormone cross-talk and its critical role in optimizing phytohormone-assisted phytoremediation.

Regarding IAA treatments (0.5–200 μM), although TG dry shoot biomass was lower than the controls (Tables S8 and S9), PFAS concentrations (ng/g) in harvestable shoots (Figures S1 and S2), and total PFAS uptake (%) (Figures 1–3), were generally higher than in control-2. In particular, treatments such as JA-1/IAA-0.5 and IAA-1-C significantly enhanced the uptake of individual PFAS compounds and Σ_8_PFAS in TG shoots. These results indicate that, in contrary to the expectation that IAA would stimulate plant growth and biomass production, foliar application of these PGRs in our study instead promoted PFAS accumulation in plant tissues.

The increased concentration of contaminants in plant tissues as a result of PGR was also reported for Cd. Shah et al. (2023) revealed Cd concentration of 294 ± 2.08 mg/kg for Cd + GA and 361 ± 4.44 mg/kg for Cd + IAA, in *Parthenium hysterophorus* (carrot grass) leaves in comparison with 170 ± 1.98 mg/kg in the control.^72^ The Cd study, however, observed higher biomass with PGR use. Thus, the response to PGR may be plant-specific.

The mechanism underlying the observed combination of reduced biomass but increased PFAS accumulation (ng/g) following foliar PGR application in this study remains unclear and warrants further investigation. One possible explanation is that PGRs influence root system architecture, including lateral root formation and root hair development, which may increase the effective root surface area available for solute uptake.^73^ Additionally, auxin signaling can alter root transport processes and regulate plant responses to environmental stresses, potentially enhancing the acquisition of dissolved contaminants under suboptimal soil conditions. Such physiological adjustments may increase contaminant accumulation or uptake efficiency even when overall plant growth is reduced.^74^

### 3.4 Effects of Selected PGRs on TG Root Biomass and PFAS Accumulation

#### 3.4.1 TG Root Biomass by Day 108

Based on results in Figure 3, several conditions, for example, IAA-1-C, IBA-10-C, GA-1/GA-0.1, GA-10/GA-0.2, and DCPAA-100/GA-5, were selected for subsequent root biomass quantification and PFAS uptake analysis. TG root biomass measurements (Table S10) revealed that IAA-1-C and IBA-10-C compared to the control-2 (6.39 ± 0.01 g) either preserved or slightly enhanced root dry mass, yielding 7.12 ± 0.13 g and 6.84 ± 0.16 g, respectively. These findings suggest that auxin-based treatments may promote robust belowground growth, likely through stimulation of adventitious rooting and lateral root development. In contrast, GA treatments at all tested concentrations resulted in reduced root biomass (∼3.2–5.7 g), falling below the control-2 (6.39 ± 0.01 g). This suggests that shoot-promoting PGRs may alter biomass allocation patterns between roots and shoots,^75^ which should be carefully considered when targeting total plant productivity. It was also observed that TG dry root biomass in control-1 was only 3.45 ± 0.29 g, at least 1.98 times lower than in control-2 (6.39 ± 0.01 g). However, this higher root biomass in control-2 with the presence of PFAS may reflect stress-induced biomass allocation rather than true growth stimulation. The plants under chemical stress often increase root development as an adaptive response to maintain water and nutrient uptake.^76,77^

#### 3.4.2 Individual and Σ_8_ PFAS Uptake (%) in TG Roots

In terms of PFAS uptake (%) by TG roots, auxin-based treatments, particularly IBA-10-C and IAA-1-C, resulted in the highest cumulative PFAS uptake in roots, with Σ_8_PFAS removal reaching 1.97 ± 0.01% and 1.65 ± 0.16%, respectively (Figure S3). These values significantly surpassed the untreated control-2 (0.79 ± 0.15%), underscoring the efficacy of low-to-moderate auxin doses in promoting PFAS uptake by roots. Among individual compounds, short-chain PFAAs such as PFHxA, and PFHpA exhibited the highest root uptake (1.97 ± 0.22% to 3.33 ± 0.33%) compared to the control-2 (0.72 ± 0.19% to 2.81 ± 0.39%), aligning with their greater solubility, reduced sorption affinity to soil particles, and improved root penetration.

It is worth noting that one-way ANOVA showed no statistically significant difference between control-2 and IBA-10 for PFBS. For PFHxS, the levels were at least 4-fold higher than control-2. For GenX, although IAA-1-C, IBA-10-C, and GA-1/GA-0.1 did not show a statistically significant difference, these conditions were still considerably higher than control-2, by at least onefold. The modest increases observed for long-chain PFOS and PFNA under IBA-10-C (0.49 ± 0.01% to 0.84 ± 0.01%) than control-2 (0.35 ± 0.03% to 0.44 ± 0.10%) suggest that increased root biomass may also marginally improve sorption of otherwise immobile species, though their translocation to shoots remains limited.^66^

A similar trend was also observed by Qian et al. (2023), who reported that long-chain PFNA, PFDA, and PFUnA exhibited significantly lower root accumulation, primarily due to higher hydrophobicity and stronger sorption to soil’s OM.^78^ GA-based treatments generally showed limited PFAS accumulation in roots, often matching or falling below control-2 levels likely due to enhanced translocation to shoots, as evidenced in Section 3.3 where GA-treated plants exhibited higher shoot accumulation than auxin-treated ones. Besides, as PFAS removal efficiency is directly proportional to dry biomass yield, the lower root biomass of TG observed in GA-treated pots (Table S10) may have further reduced their overall remediation potential. The tested PGRs also enhanced the concentrations of individual PFAS and Σ_8_PFAS (ng/g) in TG roots, as given in Text S5 and Figure S4.

### 3.5 Cross-Species Evaluation of PGR-Assisted PFAS Uptake using Alfalfa

#### 3.5.1 Growth of Alfalfa Exposed to Selected PGRs

To study the potential applicability of PGR-assisted stimulation of PFAS uptake, alfalfa was cultivated under foliar application of selected PGRs—IAA, IBA, and GA—each applied at 1, 10, or 100 μM concentrations, and evaluated for shoot and root biomass accumulation (Table S11). Given the well-known plant growth-promoting effects of auxin- and GA-based PGRs and considering that their responses may vary among plant species, these conditions were selected to be consistent with those applied to TG. Among these treatments, IBA-100, GA-1, and GA-100, produced the highest total biomass (shoots + roots) of 1.33 ± 0.21, 1.29 ± 0.28, and 1.43 ± 0.15 g, respectively, notably exceeding the control-2 (1.06 ± 0.13 g) and control-1 (1.00 ± 0.25 g) by 54 days. These results support the potential for cross-species applicability of PGR treatments to enhance plant growth in PFAS-contaminated soil.

#### 3.5.2 Individual and Σ_8_PFAS Uptake (%) of Alfalfa Exposed to Selected PGRs

As shown in Figure 4, PFAS uptake in alfalfa roots and shoots exhibited compound-specific and dose-dependent patterns. Among the treatments, IBA at 100 μM (IBA-100) enhanced uptake across PFAS of varying chain lengths and functionalities, except for PFNA. According to one-way ANOVA, the highest removal was observed for GenX (12.74 ± 0.07%) compared to control-2 (7.36 ± 0.09%), PFBS (10.33 ± 0.09%) compared to control-2 (6.28 ± 0.12%), and PFHxA (5.54 ± 0.01%) compared to control-2 (3.89 ± 0.07%). For the relatively hydrophobic PFNA, IBA-10 achieved a higher removal of 0.15 ± 0.01%, compared to control-2 (0.04 ± 0.00%). In contrast, GA at all tested concentrations (1–100 μM) showed only moderate ΣPFAS removal (3.13 ± 0.29% to 3.34 ± 0.34%) relative to the control-2 (2.56 ± 0.03%). The ΣPFAS uptake by alfalfa roots under PGR treatments ranged from 0.03 ± 0.01% to 0.18 ± 0.01%, compared to 0.14 ± 0.02% in control-2.

**Figure 4:**
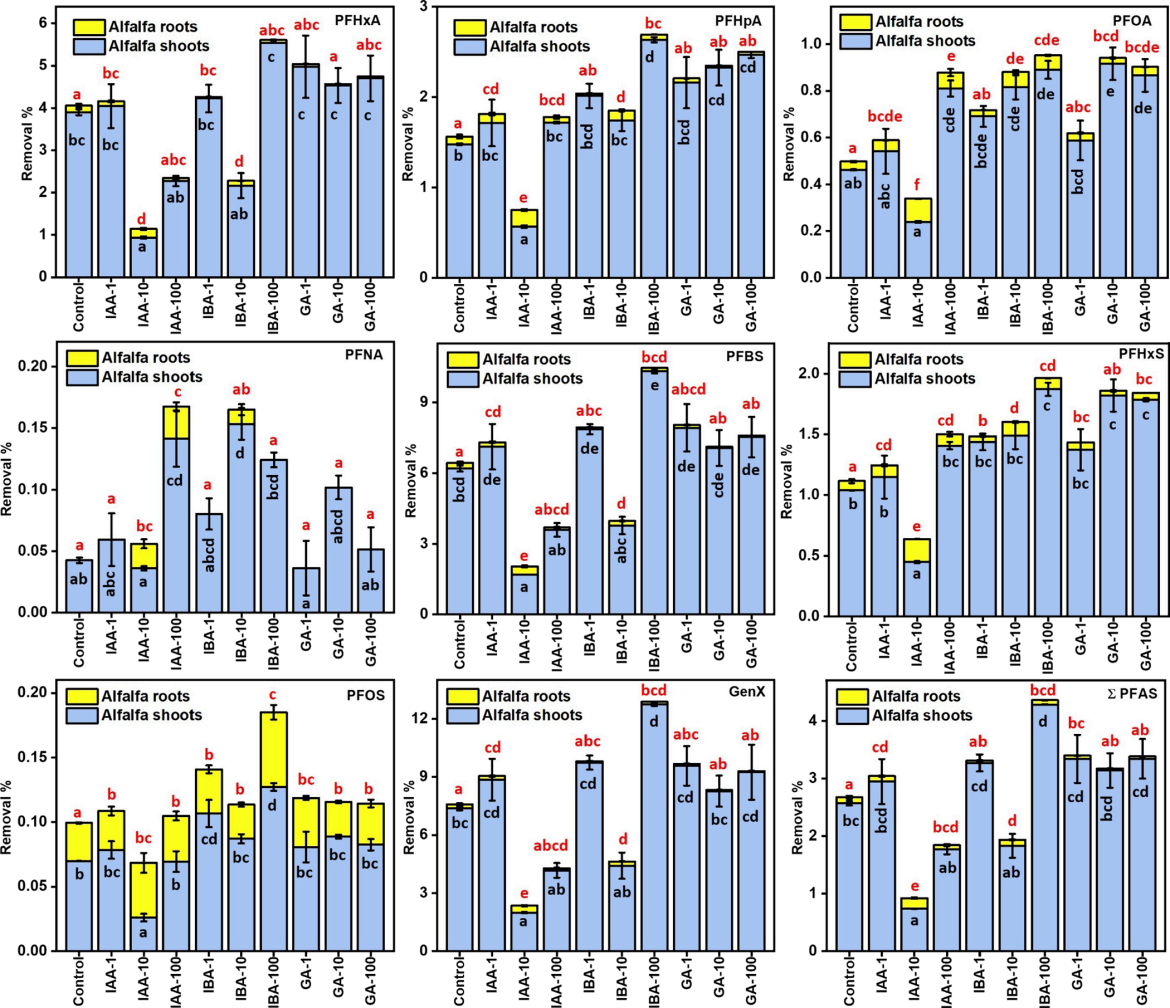
Cross-species evaluation of PGR-assisted PFAS uptake: removal (%) of individual and ∑PFAS by alfalfa shoots and roots following foliar application of selected PGRs (IAA, IBA, and GA) at 1, 10, and 100 μM (*n* = 3). Statistical significance was evaluated using one-way ANOVA followed by Tukey’s Honestly Significant Difference (HSD) test. Letters in black and red represent significant differences among the treatments with alfalfa shoots and roots, respectively (*p* < 0.05).

The long-chain PFAS, including PFOA, PFNA, and PFOS, were primarily retained in the roots with minimal translocation to shoots, reaffirming previous observations that root sorption and limited xylem mobility constrain their systemic transport.^66^ The effects of PGRs on the concentrations of individual PFAS and Σ_8_PFAS (ng/g) in alfalfa shoots and roots are discussed in Text S6 and Figure S5. Moreover, Σ_8_PFAS shoot accumulation percentages in alfalfa (Figure 4) were compared with those in TG at the first cut (Figure 3), since both plants were harvested at the same time (day 54). The key observation is that Σ_8_PFAS accumulation in alfalfa was 3- to 4-fold higher than in TG across all tested treatments. For example, under IAA-1, alfalfa shoots showed Σ_8_PFAS accumulation of 2.94 ± 0.39% compared to 1.22 ± 0.10% in TG shoots. A similar trend was evident even in the control-2, where alfalfa reached ΣPFAS accumulation of 2.56 ± 0.03% versus 0.84 ± 0.02% in TG on day 54. These differences can be attributed to plant physiology: alfalfa’s deep taproot and higher transpiration likely promoted rapid upward translocation of contaminants such as PFAS, whereas TG’s shallow fibrous roots may not be beneficial for PFAS translocation.

Apart from different plant physiology, a direct comparison of PGR dose effect between the two species may not be entirely relevant since the cumulative mass of PGRs applied to alfalfa was ∼6.7 times greater than that applied to TG during the first 54 days (Tables S2 and S3). Nonetheless, the observed correlation between increased biomass and ΣPFAS uptake in alfalfa, alongside similar responses observed with TG, supports the dual positive role of PGRs in enhancing both plant productivity and contaminant phytoextraction.^79,80^ This cross-species evaluation confirms that the application of PGRs is an effective strategy for enhancing PFAS uptake across different plant systems, underscoring its potential for broader application in field-scale phytoremediation.

## 4 Environmental Implications and Future Perspectives

This study presents a promising and interesting experimental framework for enhancing PFAS phytoextraction through the foliar application of PGRs, including IAA, IBA, and GA. The observed increase in shoot biomass and improved accumulation of short-chain PFAS such as PFHxA, PFBS, and GenX and long-chain PFHpA demonstrate the potential of PGRs to address key limitations of conventional phytoremediation, namely, low contaminant translocation and insufficient aboveground accumulation. Notably, the ability to stimulate PFAS uptake via agriculturally relevant PGRs may offer a practical route for application in managing PFAS impacted agricultural landscapes. The approach was proven valid for both Timothy grass and alfalfa.

Despite these encouraging findings, several limitations must be acknowledged. First, long-chain PFOA, PFNA, and PFOS remained primarily in the roots, exhibiting poor translocation to harvestable biomass. This points to the need for other approaches that could improve upward translocation from plant roots to shoots. Second, in order to maximize the PGR’s positive effect toward increasing PFAS uptake, the underlying mechanisms must be elucidated. Investigations aiming to understand PGR’s influence over PFAS root-to-shoot transport, membrane permeability, and transporter activity need to be performed. Third, throughout literature on PGRs, the majority of studies reported concentration of PGRs tested, while the volume of each PGR spray was not given. This creates difficulty in comparing the PGRs across different investigations and different plant species. Additionally, different studies use different frequency in applying PGR sprays, which makes any attempt in comparing the results even more impossible. Fourth, although the enhancing effect on PFAS uptake was observed in both TG and alfalfa, the best performing PGR and its dose are different between these two plant species. Given complex and different physiology among different plant species, this may seem reasonable. However, it may reveal the fact that the exact PGR effect on PFAS uptake is plant-dependent. Fifth, spiked PFAS experiments conducted in this greenhouse study may overestimate uptake and removal efficiencies compared to naturally contaminated soils. Spiking ensures uniform PFAS distribution and detectable concentrations, which facilitates controlled investigation of PGR effects on plant uptake. However, in naturally contaminated soils, PFAS may be unevenly distributed, bound to soil components or other cocontaminants, or present at lower phytoavailable concentrations, potentially reducing plant uptake. Future studies using real contaminated soils in field trials are warranted to validate the applicability of these findings under environmentally realistic conditions. Sixth, before PGRs can be used in the field, comprehensive cost-benefit analysis using field-scale relevant parameters will need to be carried out. Regardless of these limitations, however, this work does open the door for investigating PGRs as a useful treatment for enhancing PFAS removal from soil.

## Supplementary Material



## Notes

Background PFAS concentration in local soil and spiked soil; total amount of PGR applied to each pot for both TG and alfalfa; chemical reagents used in this study; physiochemical properties of PFAS examined in this study; analytical method details for PFAS quantifications via LC-MS/MS; QC; greenhouse growth progression of TG and alfalfa under PFAS-contaminated conditions and PGR treatments over time; dry weight of plant tissues under various PGR treatments; and PFAS concentration in both TG and alfalfa shoots and roots (PDF)
