# Supplementary material for: Enhancement of Per- and Polyfluoroalkyl Substance Uptake from Contaminated Soil by Applying Plant Growth Regulators: Screening and Cross-Species Evaluation
Source: ACS ES T Eng. 2026 Jun 5;6(7):1950–61. doi: 10.1021/acsestengg.6c00068 (PMC13366583; doi:10.1021/acsestengg.6c00068)
Supplement: Supplementary file 1 [file ee6c00068_si_001.pdf]

Supplementary Material

Enhancement of PFAS Uptake from Contaminated Soil by Applying Plant Growth Regulators: Screening and Cross-Species Evaluation

Aswin Kumar Ilango [a,*], Oya Aydin Urucu [a,b], Madhav Kharel [a], and Yanna Liang [a]

[a] Department of Environmental and Sustainable Engineering, University at Albany, State University of New York, Albany, New York 12222, United States

[b] Department of Chemistry, Marmara University Faculty of Sciences, Istanbul 34722, Turkey

* Corresponding authors:

Aswin Kumar Ilango (A.K. Ilango); Yanna Liang (Y. Liang)

Email: ailango@albany.edu; yliang3@albany.edu

Address: 1400 Washington Avenue, Albany, New York 12222, United States

Contents

Tables

Table S1. Background PFAS concentrations in Local soil and spiked soil-------------------- S4

Table S2. Amount (µg) and volume (mL) of PGR applied to TG from day 0 to day 108--- S5 and S6

Table S3. Amount (µg) and volume (mL) of PGR applied to Alfalfa growth period 0-54 days-- S7

Table S4. Chemical reagents used in this study--- S8

Table S5. Physiochemical properties of PFAS examined in this study------------------------ S9 and S10

Table S6. Dynamic multiple reaction monitoring transitions, limit of detection (LOD) and limit of quantification (LOQ) of the PFAS tested in this study----------------------------- S13

Table S7. Quality control (QC): percent recovery of ^{13}C labeled and native PFAS in different matrices--- S13

Table S8. Dry weight of TG shoots under various PGR treatments at the first harvest (Day 54)--- S14

Table S9. Dry weight of TG shoots under various PGR treatments at the second harvest (Day 108)--- S14 and S15

Table S10. Dry weight of TG roots under various PGR treatments---------------------------- S15

Table S11. Dry weight of Alfalfa shoots and roots under various PGR treatments (Day 54)--- S15

Texts

Text S1. Equations used for calculating PFAS extraction efficiency, concentration, and removal efficiency-- S11

Text S2. Analytical method details for PFAS quantifications via LC-MS/MS--------------- S12

Text S3. Effects of PGRs on individual and ΣPFAS concentrations (ng/g) in TG shoots in 54 days--- S16

Text S4. Individual PFAS concentrations (ng/g) in TG shoots day 55-108------------------ S18

Text S5. Individual and Σ_8PFAS concentrations (ng/g) in TG roots--------------------------- S21

Text S6. PFAS concentration (ng/g) in Alfalfa --- S23 to S25

Figures

Figure S1. Concentration (ng/g) of individual and Σ_8PFAS from soil by TG during the first harvest (Day 54) under foliar application of various PGRs------------------------------- S17

Figure S2 Concentration (ng/g) of individual and Σ_8PFAS from soil by TG shoots during the second harvest (Day 108) following selected foliar application of PGRs-------------- S19

Figure S3. Root uptake of PFAS by TG under different PGR treatments------------------- S20

Figure S4. Concentration (ng/g) of individual and Σ_8PFAS from soil by TG roots under different PGR treatments-- S22

Figure S5. Concentration (ng/g) of individual and ∑PFAS by Alfalfa shoots and roots following foliar application of selected PGRs (IAA, IBA, and GA) at 1, 10, and 100 µM-- S23

Table S1. Background PFAS concentrations in Local soil and spiked soil

PFAS tested	PFAS concentration (μg/kg)	
	Local soil	Spiked soil
PFHxA	ND	97.0 ± 11.5
PFHpA	ND	93.2 ± 7.97
PFOA	0.166 ± 0.01	96.6 ± 9.97
PFNA	0.116 ± 0.01	99.2 ± 9.41
PFBS	ND	101 ± 14.8
PFHxS	ND	100 ± 13.5
PFOS	0.220 ± 0.01	87.1 ± 10.6
GenX	ND	94.2 ± 13.5

Note: ND: non-detected

Table S2. Amount (µg) and volume (mL) of PGR applied to TG from day 0 to day 108. IAA-1 means IAA was sprayed at 1 µM. JA-10/IAA-200 means that the TG pots sprayed with JA at 10 µM from Day 1 to 54 were subsequently treated with IAA at 200 µM from Day 55-108.

	Pot no.	Treatment #	PGR-concentration (µM)	Replicates	PFAS spiked	Amount of PGR sprayed (µg)		
						Spray on Day 27, and 34, Vol: 2.5 mL	Spray on Day 41 and 48, Vol: 5 mL	Total amount of PGR sprayed (µg)
Prior to first cut (Day 0 to day 54)	1	Control-1	N/A	3	No	N/A	N/A	N/A
	2	Control-2	N/A	3	Yes	N/A	N/A	N/A
	3	IAA-1	1	3	Yes	0.44	0.88	2.60
	4	IAA-10	10	3	Yes	4.38	8.76	26.2
	5	IAA-100	100	3	Yes	43.8	87.6	263
	6	IBA-1	1	3	Yes	0.51	1.02	3.03
	7	IBA-10	10	3	Yes	5.08	10.1	30.5
	8	IBA-100	100	3	Yes	50.8	102	305
	9	GA-1	1	3	Yes	0.87	1.73	5.18
	10	GA-10	10	3	Yes	8.66	17.3	51.9
	11	GA-100	100	3	Yes	86.6	173	520
	12	JA-1	1	3	Yes	0.53	1.05	3.14
	13	JA-10	10	3	Yes	5.26	10.5	31.5
	14	JA-100	100	3	Yes	52.6	105	315
	15	DCPAA-1	1	3	Yes	0.55	1.11	3.30
	16	DCPAA-10	10	3	Yes	5.53	11.1	33.1
	17	DCPAA-100	100	3	Yes	55.3	111	277
	18	NAA-1	1	3	Yes	0.47	0.93	6.97
	19	NAA-10	10	3	Yes	4.66	9.31	69.8
	20	NAA-100	100	3	Yes	46.6	93.1	279

	Pot no.	Treatment #	PGR-concentration (µM)	Replicates	PFAS spiked	Amount of PGR sprayed (µg)			
						Spray on Day 91, 97 and 105, Vol: 25 mL	Second spray (Day 98) Vol: 25 mL	Third spray (Day 105) Vol: 25 mL	Total amount of PGR sprayed (µg)
Prior to second cut (Day 55 to day 108)	1	Control-1	N/A	3	No	N/A	N/A	N/A	N/A
	2	Control-2	N/A	3	Yes	N/A	N/A	N/A	N/A
	3	JA-1/IAA-0.5	0.5	3	Yes	2.19	2.19	2.19	6.55
	4	IAA-1-C	1	3	Yes	4.38	4.38	4.38	13.1
	5	IAA-10-C	10	3	Yes	43.8	43.80	43.80	131
	6	IAA-100-C	100	3	Yes	438	437.98	437.98	1310
	7	JA-10/IAA-200	200	3	Yes	876	875.95	875.95	2630
	8	IBA-1/IBA-5	5	3	Yes	25.4	25.41	25.41	76.2
	9	IBA-10-C	10	3	Yes	50.8	50.81	50.81	152
	10	IBA-100/IBA-15	15	3	Yes	76.2	76.22	76.22	229
	11	JA-100/IBA-20	20	3	Yes	102	101.62	101.62	305
	12	DCPAA-1/IBA-50	50	3	Yes	254	254.05	254.05	762
	13	GA-1/GA-0.1	0.1	3	Yes	0.87	0.87	0.87	2.58
	14	GA-10/GA-0.2	0.2	3	Yes	1.73	1.73	1.73	5.19
	15	GA-100/GA-0.5	0.5	3	Yes	4.33	4.33	4.33	13.0
	16	DCPAA-10/GA-1	1	3	Yes	8.66	8.66	8.66	26.0
	17	DCPAA-100/GA-5	5	3	Yes	43.3	43.30	43.30	130
	18	NAA-1/IBA-10a	10	3	Yes	50.8	50.81	50.81	152
	19	NAA-10/IBA-10b	10	3	Yes	50.8	50.81	50.81	152
	20	NAA-100/IBA-10c	10	3	Yes	50.8	50.81	50.81	152

Note: PGR: Plant growth regulators; IAA: Indole-3-acetic acid; IBA: Indole-3-butyric acid; gibberellic acid (GA); jasmonic acid (JA); DCPAA: 2,4-dichlorophenoxyacetic acid; and NAA: naphthaleneacetic acid. N/A: not applicable.

Table S3. Amount (µg) and volume (mL) of PGR applied to Alfalfa growth period 0-54 days. IAA-1 means IAA was sprayed at 1 µM.

	Pot no.	Treatment #	PGR-concentration (µM)	Replicates	PFAS spiked	Amount of PGR sprayed (µg)	
						Mass of spray on Day 27, 34, 41 and 48	Total amount of PGR sprayed (µg)
**Prior to first cut (Day 0 to day 54)**	1	Control-1	N/A	3	No	N/A	N/A
	2	Control-2	N/A	3	Yes	N/A	N/A
	3	IAA-1	1	3	Yes	4.38	17.5
	4	IAA-10	10	3	Yes	43.8	175
	5	IAA-100	100	3	Yes	438	1750
	6	IBA-1	1	3	Yes	5.08	20.3
	7	IBA-10	10	3	Yes	50.8	203
	8	IBA-100	100	3	Yes	508	2030
	9	GA-1	1	3	Yes	8.66	34.6
	10	GA-10	10	3	Yes	86.6	346
	11	GA-100	100	3	Yes	866	3460

Note: PGR: Plant growth regulators; IAA: Indole-3-acetic acid; IBA: Indole-3-butyric acid; and gibberellic acid (GA). N/A: not applicable

Table S4. Chemical reagents used in this study.

No.	Chemical name	Grade/purity	Procured detail
1.	Indole-3-butyric acid	98%	Thermo scientific
2.	Indole-3-acetic acid	98%	Thermo scientific
3.	Gibberellic acid	99%	Thermo scientific
4.	Jasmonic acid	>98%	Thermo scientific
5.	α-Naphthaleneacetic acid	98%	Research Products International
6.	2,4-Dichlorophenoxyacetic acid	98%	Thermo scientific
7.	Perfluorohexanoic acid	≥98%	Fisher scientific
8.	Perfluoroheptanoic acid	≥98%	Fisher scientific
9.	Perfluorooctanoic acid	≥96%	Sigma-Aldrich
10.	Perfluorononanoic acid	≥98%	Oakwood chemicals
11.	Potassium perfluorobutanesulfonate	≥98%	Fisher scientific
12.	Perfluorohexanesulfonic acid potassium salt	≥98%	Fisher scientific
13.	Heptadecafluorooctanesulfonic acid potassium salt	≥98%	Sigma-Aldrich
14.	Undecafluoro-2-methyl-3-oxahexanoic acid	≥97%	SynQuest Laboratories
15.	Perfluoro-n-[1,2,3,4,6-$^{13}C_5$]hexanoic acid	≥98%	Wellington Laboratories Inc.
16.	Perfluoro-1-[1,2,3-$^{13}C_3$]hexanesulfonic acid	≥98%	Wellington Laboratories Inc.
17.	Perfluoro-n-[1,2-$^{13}C_2$]octanoic acid	≥98%	Wellington Laboratories Inc.
18.	Sodium perfluoro-1[1,2,3,4-$^{13}C_4$]octanesulfonate	≥98%	Wellington Laboratories Inc.
19.	Sodium hydroxide	Certified ACS	Fisher scientific
20.	Tetrabutylammonium hydrogen sulfate	99%	Thermo scientific
21.	Sodium carbonate	98%	Thermo scientific
22.	Tert-Butyl methyl ether	99%	Fisher scientific
23.	Bond Elut PFAS WAX cartridge	150 mg, 6mL	Agilent technologies
24.	Ammonium hydroxide	28-30%	Fisher scientific
25.	Methanol	LCMS/HPLC	Fisher scientific
26.	Ammonium acetate	>99%	Fisher scientific
27.	Acetic acid	>99%	Sigma-Aldrich
28.	Water	LCMS	Fisher scientific

Table S5. Physiochemical properties of PFAS examined in this study.

No.	Category	Compound name	Chemical structure	Chemical formula	Mol. weight (g/mol)	S_w (25 °C) (g/L)	pK_a (25 °C)
1.	Short-chain PFCA	Perfluorohexanoic acid (PFHxA)	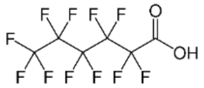	$C_6HF_{11}O_2$	314	15.7 [1]	-0.16 [2]
2.	Short-chain PFCA	Perfluoroheptanoic acid (PFHpA)	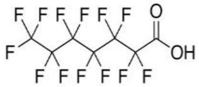	$C_7HF_{13}O_2$	364	3.65×10^{-3} [3]	−2.29[3]
3.	Long-chain PFCA	Perfluorooctanoic acid (PFOA)	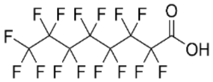	$C_8HF_{15}O_2$	414	3.4 [1]	-0.2[2]
4.	Long-chain PFCA	Perfluorononanoic acid (PFNA)	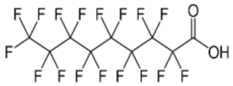	$C_9HF_{17}O_2$	464	6.25×10^{-2} [3]	-0.21[3]
5.	Short-chain PFSA	Potassium perfluorobutane sulfonate (PFBS)	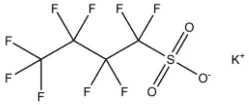	$C_4F_9O_3SK$	338	46.2 [4]	0.14[2]
6.	Long-chain PFSA	Perfluorohexanesulfonic acid potassium salt (PFHxS)	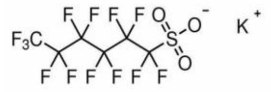	$C_6F_{13}KO_3SK$	438	2.3[5]	0.14[2]
7.	Long-chain PFSA	Heptadecafluorooctane sulfonic acid potassium salt (PFOS)	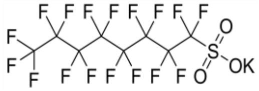	$C_8HF_{17}KO_3SK$	538	0.57[1]	-3.27[6]

8.	PFOA alternative	Undecafluoro-2-methyl-3-oxahexanoic acid (GenX)	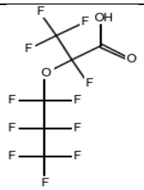	$C_6HF_{11}O_3$	330.05	N/A	2.84[7]
9.	Mass labeled	perfluoro-n-[1,2,3,4,6-$^{13}C_5$]hexanoic acid ($^{13}C_5$-PFHxA)	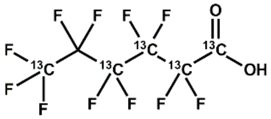	$^{13}C_5C_1HF_{11}O_2$	319.02	N/A	N/A
10.	Mass labeled	Sodium perfluoro-1-[1,2,3-$^{13}C_3$]hexanesulfonate ($^{13}C_3$-PFHxS)	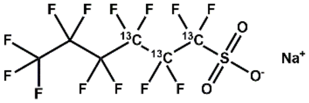	$C_6F_{13}NaO_3S$	425.08	N/A	N/A
11.	Mass labeled	perfluoro-n-[1,2,3,4-$^{13}C_4$]octanoic acid ($^{13}C_4$-PFOA)	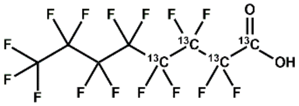	$^{13}C_4C_4HF_{15}O_2$	418.04	N/A	N/A
12.	Mass labeled	perfluoro-n-[1,2,3,4-$^{13}C_4$]octanesulfonic acid ($^{13}C_4$-PFOS)	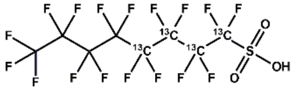	$C_4(^{13}C)_4HF_{17}O_3S$	526.08	N/A	N/A

Note: Solubility in water (S_w); Dissociation constant ($_pK_a$); N/A: Data not available

Text S1. Equations used for calculating PFAS extraction efficiency, concentration, and removal efficiency.

The extraction efficiency (%) of individual and Σ_8PFAS compounds in the soil was calculated as the ratio of the total extractable mass of PFAS from soil (ng) to the total mass of PFAS spiked to the soil (ng), multiplied by 100 which is given as eqns. (S1) as given below

$$\text{Extraction efficiency (\%)} = \frac{\text{Mass of PFAS extracted from soil (ng)}}{\text{Mass of PFAS spiked to soil (ng)}}\ x\ 100 \qquad \text{(S1)}$$

PFAS concentration (ng/g) of individual and Σ_8PFAS compounds in plant shoots and roots (ng/g dry weight) were calculated by multiplying the PFAS concentration measured in the extract (ng/L) by the final extract volume (L), and dividing by the dry mass of the plant tissue sample (g), as shown in eqns. (S2) below.

$$\text{PFAS concentration (ng/g)} = \frac{C_{extract}\ X\ V_{extract}}{m_{sample}} \qquad \text{(S2)}$$

where, $C_{extract}$ is PFAS concentration measured in the extract (ng/L) from LC-MS/MS, $V_{extract}$ is final volume of extract (L), and m_{sample} is dry mass of plant tissue used for extraction (g), respectively.

The removal efficiency (%) of individual and Σ_8PFAS compounds by the TG and Alfalfa was calculated as the ratio of the total PFAS mass accumulated in plant tissues ($C_{plants} \times \text{total } m_{plants}$) to the total PFAS mass initially spiked to the soil, multiplied by 100, as shown in eqns. (S3).

$$\text{Removal efficiency (\%)} = \frac{C_{plants}\ X\ \text{total } m_{plants}}{m_{soil}}\ \text{x}\ 100 \qquad \text{(S3)}$$

where, C_{plants} is PFAS concentration in plant tissues (ng/g), m_{plants} is total dry mass of harvested plants (g) and m_{soil} is total PFAS mass initially spiked to soil (ng).

Text S2. Analytical method details for PFAS quantifications via LC-MS/MS

Eight PFAS were quantified in the sample extracts, including perfluorocarboxylic acids (PFCAs: C_6-C_9), perfluorosulfonic acids (PFSAs: C_4, C_6, and C_8), and GenX. To account for instrumental drift and matrix effects, non-extracted internal standards (NIS), perfluoro-n-[1,2,3,4-$^{13}C_4$]octanoic acid ($^{13}C_4$-PFOA) and perfluoro-n-[1,2,3,4-$^{13}C_4$]octanesulfonic acid ($^{13}C_4$-PFOS), were spiked into all samples at a concentration of 1 μg/L prior to analysis [8,9].

Chromatographic separation was performed using two Agilent Eclipse Plus C18 columns: an analytical column (ZORBAX, 3 × 50 mm, 1.8 μm) and a delay column (4.6 × 50 mm, 3.5 μm), both maintained at 50 °C. The binary mobile phase consisted of 5 mM ammonium acetate in water (solvent A) and 95% methanol (solvent B). A gradient elution was applied at a flow rate of 0.5 mL/min, decreasing solvent A from 70% to 0% over 8 min, holding at 0% for 4 min, followed by a 12-min re-equilibration to initial conditions.

Detection was conducted using an Agilent 6470 Triple Quadrupole LC-MS/MS system with Jet Stream electrospray ionization (ESI) in negative ion mode, operated in dynamic multiple reaction monitoring (dMRM) mode for quantification [9-11].

Table S6. Dynamic multiple reaction monitoring transitions, limit of detection (LOD) and limit of quantification (LOQ) of the PFAS tested in this study.

Compound name	Retention time (min)	Precursor ion	Quantification ion mass	Confirmation ion mass	LOD (ng/L)	LOQ (ng/L)
PFHxA	4.67	313	269	119	2.44	39.1
PFHpA	5.63	363	319	169	4.88	39.1
PFOA	6.3	413	369	169	1.22	78.1
PFNA	6.8	463	419	169	4.88	78.1
PFBS	3.6	299	99	80	2.44	39.1
PFHxS	5.7	399	99	80	1.22	39.1
PFOS	6.84	499	99	80	1.22	39.1
GenX	4.9	285	185	169	4.88	39.1
$^{13}C_5$-PFHxA	4.911	315	269	119	2.44	39.1
$^{13}C_5$-PFHxS	5.94	402	99	80	2.44	39.1

Table S7. Quality control (QC): percent recovery of 13C labeled and native PFAS in different matrices. biomass matrix.

	QC	Method blank (%)	Matrix blank (%)	LLOPR (%)	MLOPR (%)
EIS	$^{13}C_5$-PFHxA	85.1	108	90.3	92.0
	$^{13}C_5$-PFHxS	83.2	101	92.4	100
Target PFAS	PFHxA	ND	ND	123	115
	PFHpA	ND	ND	123	127
	PFOA	ND	ND	128	124
	PFNA	ND	ND	97.3	109
	PFBS	ND	ND	118	120
	PFHxS	ND	ND	107	123
	PFOS	ND	ND	108	123
	GenX	ND	ND	121	110

note: The PFAS with concentrations below MDLs were reported as non-detected (ND), LLOPR - low level ongoing precision and recovery standards, and MLOPR - mid-level OPR.

Table S8. Dry weight of TG shoots under various PGR treatments at the first harvest (Day 54).

Treatment #	TG dry shoots (g)
Control-1	2.01 ± 0.04
Control-2	2.04 ± 0.03
IAA-1	2.06 ± 0.18
IAA-10	1.52 ± 0.21
IAA-100	1.74 ± 0.08
IBA-1	2.06 ± 0.21
IBA-10	3.40 ± 0.27
IBA-100	1.94 ± 0.14
GA-1	1.17 ± 0.27
GA-10	1.64 ± 0.09
GA-100	1.85 ± 0.72
JA-1	1.82 ± 0.43
JA-10	1.63 ± 0.16
JA-100	1.61 ± 0.19
DCPAA-1	1.29 ± 0.23
DCPAA-10	1.47 ± 0.33
DCPAA-100	1.25 ± 0.28
NAA-1	1.43 ± 0.45
NAA-10	1.97 ± 0.05
NAA-100	1.80 ± 0.12

Table S9. Dry weight of TG shoots under various PGR treatments at the second harvest (Day 108).

Treatment #	TG dry shoots (g)
Control-1	2.52 ± 0.12
Control-2	2.46 ± 0.20
JA-1/IAA-0.5	2.35 ± 0.08
IAA-1-C	1.88 ± 0.03
IAA-10-C	2.37 ± 0.14
IAA-100-C	2.07 ± 0.19
JA-10/IAA-200	2.23 ± 0.20
IBA-1/IBA-5	2.26 ± 0.31
IBA-10-C	2.73 ± 0.06
IBA-100/IBA-15	1.26 ± 0.15
JA-100/IBA-20	2.39 ± 0.13
DCPAA-1/IBA-50	1.95 ± 0.11
GA-1/GA-0.1	2.42 ± 0.01
GA-10/GA-0.2	2.77 ± 0.25
GA-100/GA-0.5	3.16 ± 0.02

DCPAA-10/GB-1	2.14 ± 0.05
DCPAA-100/GB-5	2.08 ± 0.01
NAA-1/IBA-10a	3.02 ± 0.17
NAA-10/IBA-10b	3.49 ± 0.10
NAA-100/IBA-10c	2.79 ± 0.21

Table S10. Dry weight of TG roots under various PGR treatments.

Treatment #	TG dry roots (g)
Control-1	3.45 ± 0.29
Control-2	6.39 ± 0.01
IAA-1-C	7.12 ± 0.13
IBA-10-C	6.84 ± 0.16
GA-1/GA-0.1	3.28 ± 0.10
GA10-GA-0.2	5.72 ± 0.18
DCPAA-100/GA-5	3.23 ± 0.31

Table S11. Dry weight of Alfalfa shoots and roots under various PGR treatments (Day 54).

Treatment #	Alfalfa dry biomass (g)		
	Shoots	Roots	Shoots + Roots
Control-1	0.71 ± 0.10	0.29 ± 0.15	1.00 ± 0.25
Control-2	0.68 ± 0.09	0.38 ± 0.04	1.06 ± 0.13
IAA-1	0.72 ± 0.10	0.38 ± 0.27	1.10 ± 0.37
IAA-10	0.28 ± 0.25	0.22 ± 0.18	0.50 ± 0.43
IAA-100	0.47 ± 0.01	0.25 ± 0.30	0.72 ± 0.31
IBA-1	0.86 ± 0.13	0.30 ± 0.12	1.16 ± 0.25
IBA-10	0.52 ± 0.14	0.20 ± 0.16	0.72 ± 0.30
IBA-100	0.80 ± 0.03	0.53 ± 0.18	1.33 ± 0.21
GA-1	0.87 ± 0.21	0.42 ± 0.17	1.29 ± 0.28
GA-10	0.82 ± 0.07	0.25 ± 0.31	1.07 ± 0.38
GA-100	1.02 ± 0.10	0.41 ± 0.05	1.43 ± 0.15

Text S3. Effects of PGRs on individual and ΣPFAS concentrations (ng/g) in TG shoots in 54 days

To evaluate the effect of PGR dose on PFAS accumulation in plants, PFAS concentrations (ng/g) in first-cut TG shoots (Day 54) were calculated and are shown in **Figure S1**. The main observation is that PGR treatments influenced the accumulation of short- and medium-chain PFCAs, whereas long-chain PFAS such as PFOA, PFNA and PFOS showed minimal response, with high variability observed across replicates. Among the PGRs tested, IAA, IBA, and GA consistently enhanced PFAS accumulation relative to the untreated controls. The effect of IAA was particularly dose-dependent: increasing IAA from 1 μM to 100 μM led to a rise in PFHxA concentration from 2354.34 ± 152.27 ng/g to 3212.75 ± 241.36 ng/g and PFHpA from 517.51 ± 34.61 ng/g to 778.66 ± 68.02 ng/g, representing at least 1.2-1.9-fold increases over the controls.

In the case of GA treatments, GA-1 increased the accumulation of several short- and medium-chain PFAS, including PFHxA, PFHpA, PFBS, and GenX, suggesting that these PGRs enhanced PFAS translocation from soil to shoots. IBA treatments (both 10 μM and 100 μM) also resulted in higher PFAS concentrations compared to controls, although the increases were generally smaller than those observed with IAA-100 or GA-1. In contrast, other PGRs exhibited a different trend: increasing their foliar concentrations from 1 μM to 100 μM led to a decrease in PFAS accumulation, indicating that the response to PGRs is both compound- and dose-dependent. These observations highlight the importance of optimizing PGR type and concentration for foliar applications to maximize PFAS uptake in TG shoots.

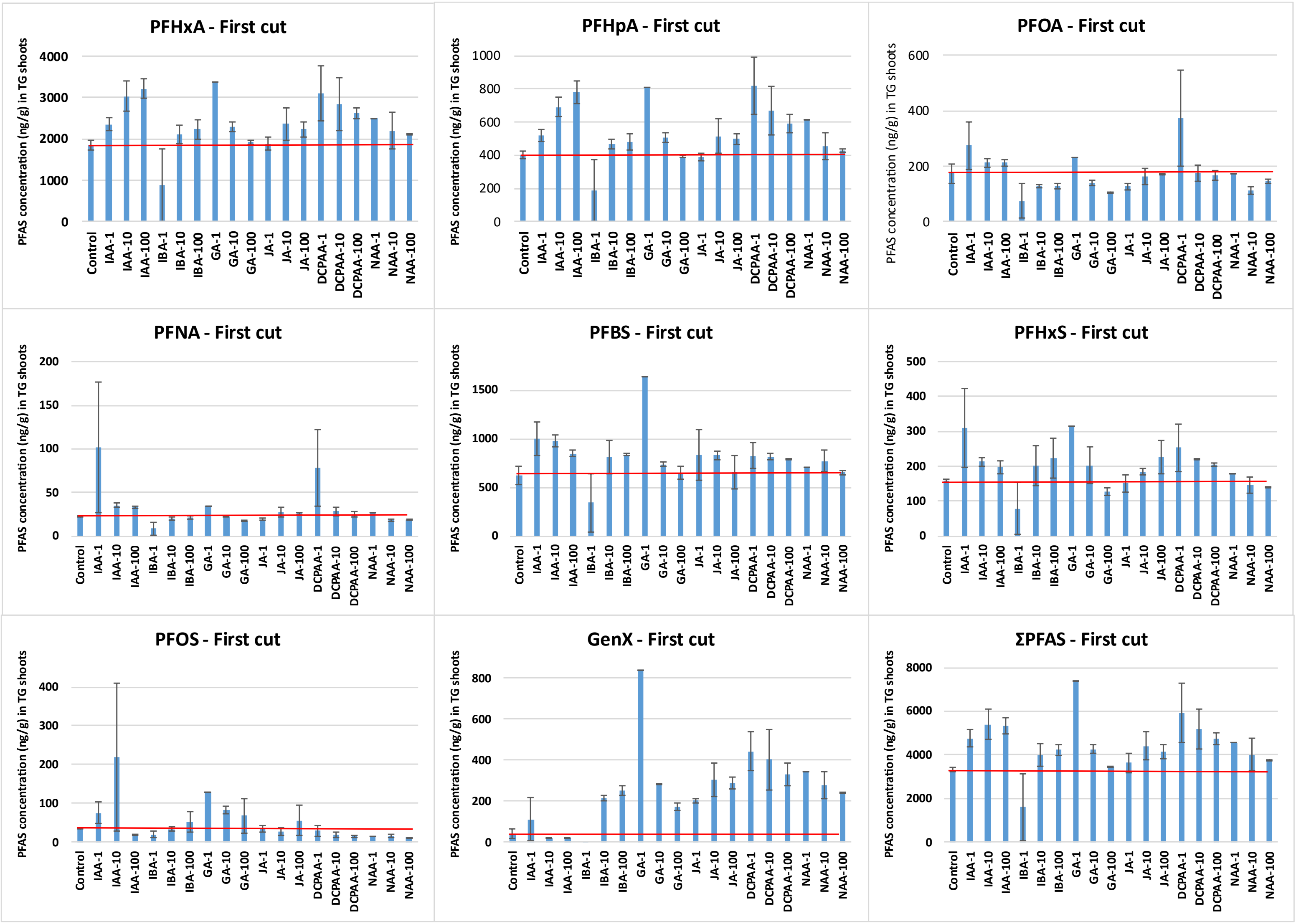

Figure S1. Concentration (ng/g) of individual and Σ_8PFAS in TG shoots during the first harvest (Day 54) under foliar application of various PGRs. Red horizontal lines indicate average removal in the control (control-2) without exposure to PGRs (n = 3).

Text S4. Individual PFAS concentrations (ng/g) in TG shoots day 55-108

The effect of selected PGR doses on PFAS accumulation (ng/g) in TG shoots at Day 108 is shown in **Figure S2**. Only a few PGR treatments resulted in increased PFAS concentrations (ng/g) in TG shoots after the 108-day growth period. For example, in the GA treatments, the 0.1 µM dose (GA-1/GA-0.1) showed elevated PFAS accumulation of 9328.26 ± 87.96 ng/g, 4108.87 ± 1.33 ng/g, and 5557.42 ± 212.50 ng/g for PFHxA, PFHpA, and PFBS, respectively. Furthermore, GA at 5 µM (DCPAA-100/GA-5) resulted in the highest PFAS accumulation for the short-chain compounds PFHxA, PFHpA, and PFBS, with concentrations of 13085.91 ± 1368.00 ng/g, 4195.70 ± 344.34 ng/g, and 5696.26 ± 485.00 ng/g, respectively, which were approximately 1.8-2.3 times higher than those observed in the control. In addition, GA at 5 µM (DCPAA-100/GA-5) showed responses similar to those of GA-1/GA-0.1 for both PFHpA and PFBS. For long-chain PFAS such as PFOA, PFNA, and PFOS, none of the GA doses demonstrated a positive effect.

For the IAA treatments, IAA-1-C led to higher PFAS concentration than IAA-100-C in TG shoots, and this trend was consistent across all analyzed PFAS compounds. Reducing the IAA dose to 0.5 µM (JA-1/IAA-0.5) or increasing it to 200 µM (JA-1/IAA-200), however, did not result in a positive effect on PFAS accumulation. It should be noted that the IAA-0.5 µM and IAA-200 µM treatments also received JA at 1 µM and 10 µM, respectively. Similar to IAA, IBA treatments did not give notable increases in PFAS concentrations in TG shoots.

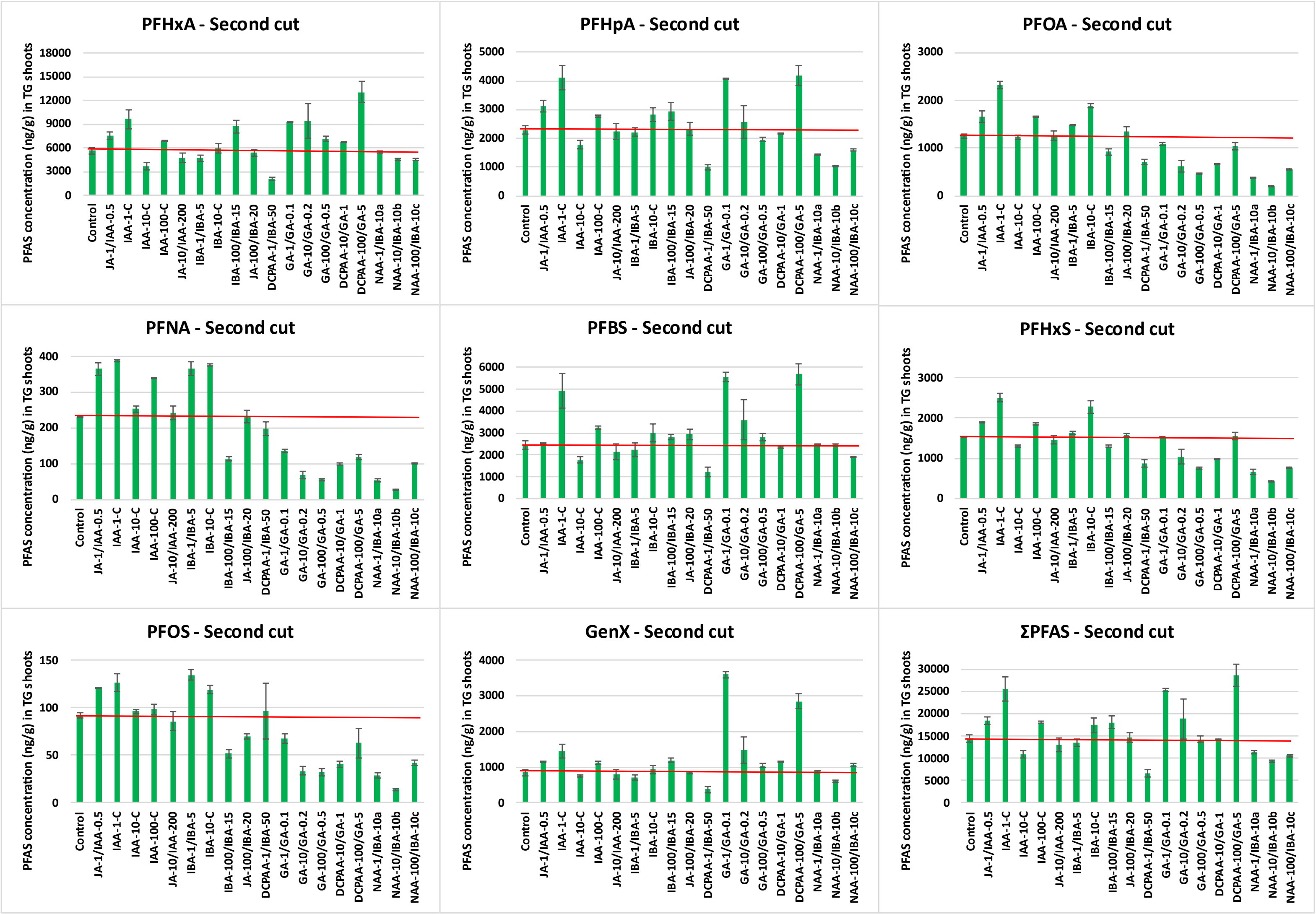

Figure S2. Concentration (ng/g) of individual and Σ_8PFAS in TG shoots during the second harvest (Day 108) following refined foliar application of PGRs. Red horizontal lines indicate average ng/g in the control (control-2) without exposure to PGRs (n = 3).

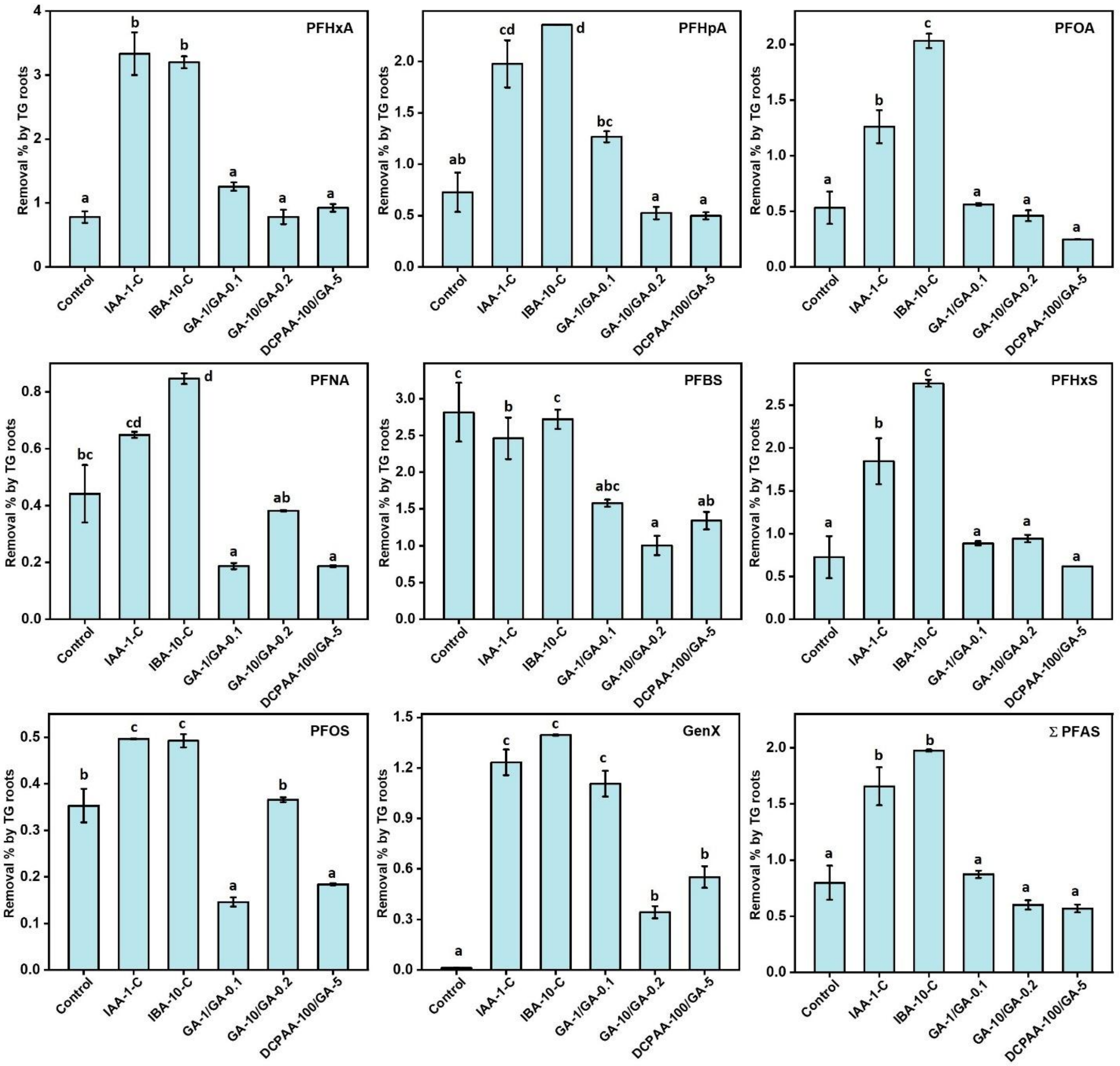

Figure S3. Root uptake of PFAS by TG under different PGR treatments. Bars represent the mean percentage removal of individual PFAS compounds by TG roots on day 108 (n = 3). Letter represents significant differences among the treatments with TG roots, respectively ($p < 0.05$).

Text S5. Individual and Σ_8PFAS concentrations (ng/g) in TG roots

Similar to what was observed for TG shoots, the tested PGRs also enhanced the individual and Σ_8PFAS concentrations (ng/g) in TG roots (**Figure S4**). In particular, IAA-1-C, IBA-10-C, and GA-1/GA-0.1 strongly promoted PFAS accumulation in the root tissues. Among the tested PFAS, PFHxA showed the highest root accumulation, reaching 468.02 ± 46.85 ng/g, 468.00 ± 13.67 ng/g, and 383.35 ± 19.77 ng/g under these treatments, respectively, compared to 121.95 ± 14.21 ng/g in the untreated control. For long-chain PFAS, IBA-10-C resulted in higher root concentrations of 297.20 ± 9.43 ng/g, 123.83 ± 2.73 ng/g, and 72.05 ± 2.08 ng/g for PFOA, PFNA, and PFOS, respectively, compared with the control (83.29 ± 22.79 ng/g, 69.04 ± 15.81 ng/g, and 55.25 ± 5.63 ng/g in the same PFAS order). GenX accumulation in the control was minimal (1.83 ± 0.53 ng/g), whereas all PGR treatments enhanced GenX accumulation, with GA-1/GA-0.1 showing the highest level (336.90 ± 23.41 ng/g). In contrast, the highly mobile short-chain PFBS did not substantially accumulate in the roots, and PGR treatments did not significantly influence its root concentration (**Figure S4**). Among the tested treatments, GA-10/GA-0.2 did not promote the accumulation of most individual PFAS (except GenX) or Σ_8PFAS.

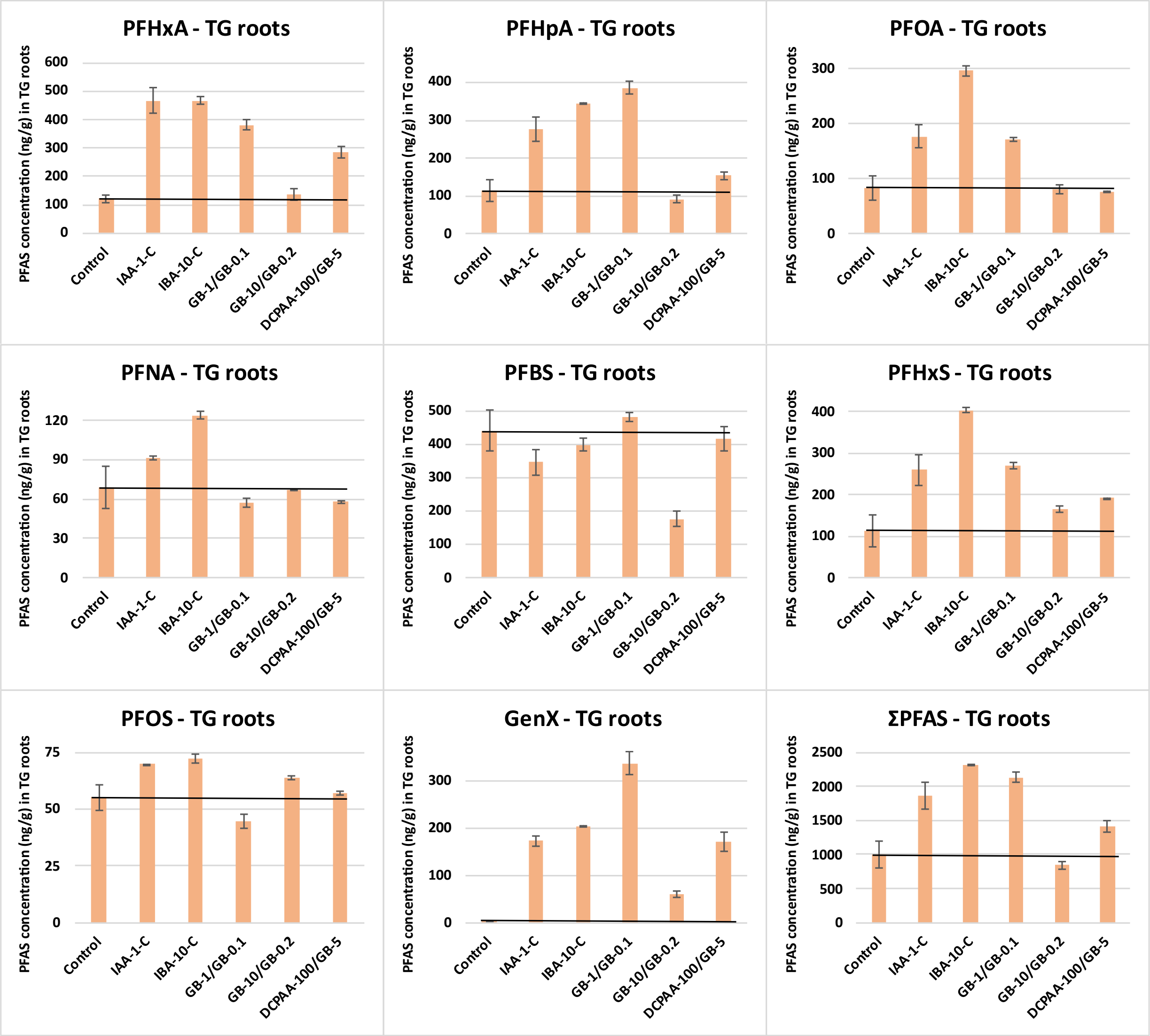

Figure S4. Concentration (ng/g) of individual and Σ_8PFAS in TG roots under different PGR treatments on day 108 (n = 3). Black horizontal lines indicate average ng/g in the control (control-2) without exposure to PGRs (n = 3).

Text S6. PFAS concentration (ng/g) in Alfalfa

The concentrations (ng/g) of individual and ΣPFAS in Alfalfa shoots and roots following foliar application of selected PGRs (IAA, IBA, and GA) at 1, 10, and 100 µM are shown in **Figure S5**. Among the tested PGRs, IAA exhibited a dose-dependent response, increasing the concentrations of long-chain PFAS, including PFOA, PFNA, PFHxS, and PFOS, as the IAA dose increased from 1 µM to 100 µM.

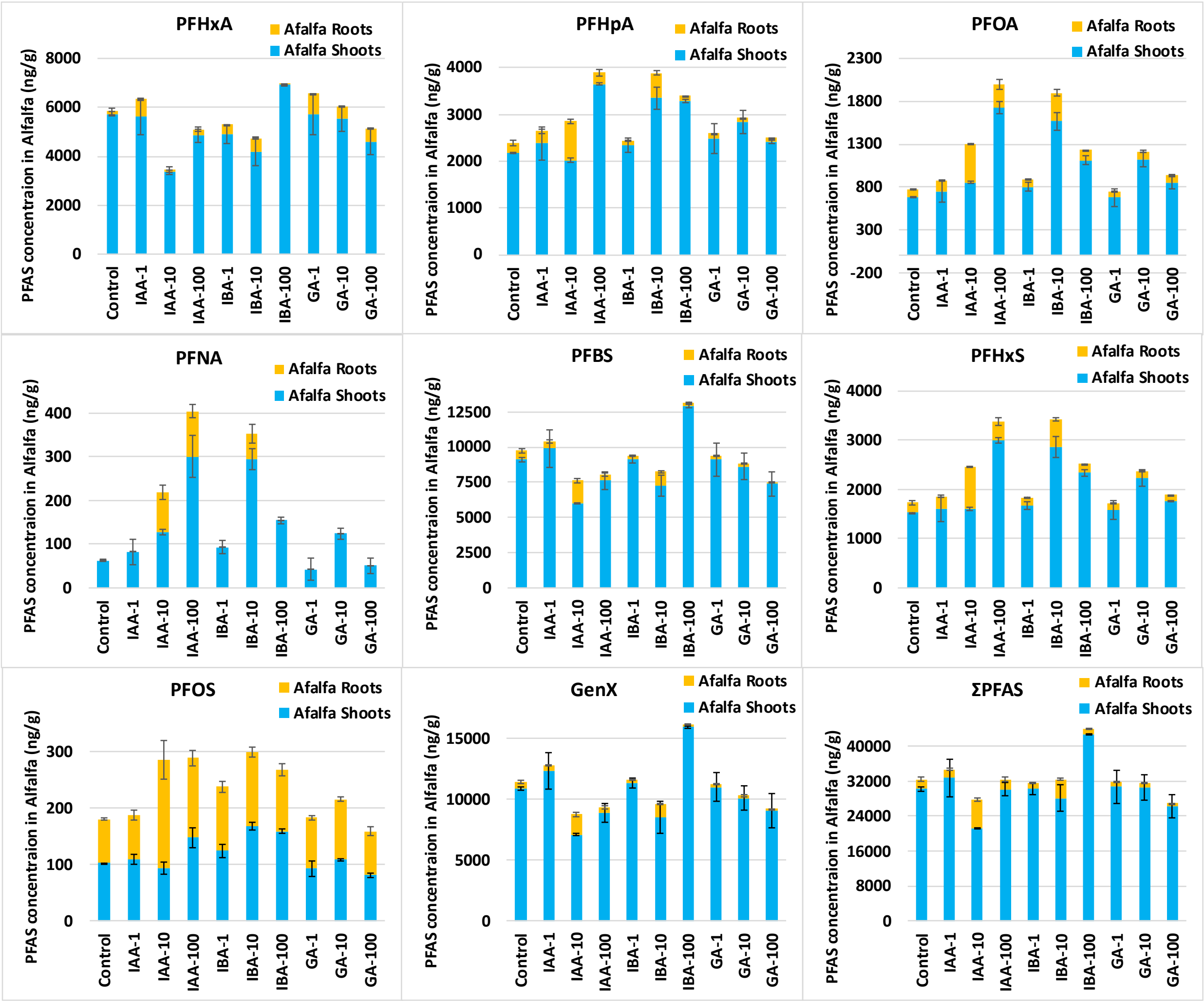

Figure S5. Concentration (ng/g) of individual and ∑PFAS in Alfalfa shoots and roots following foliar application of selected PGRs (IAA, IBA, and GA) at 1, 10, and 100 µM (n = 3).

Specifically, in Alfalfa shoots, PFOA concentrations increased from 751.79 ± 133.86 ng/g at 1 μM IAA to 1723.67 ± 72.97 ng/g at 100 μM IAA, while PFNA concentrations increased from 82.37 ± 29.67 ng/g to 300.96 ± 48.47 ng/g over the same dose range. In comparison, the control treatment showed PFOA and PFNA concentrations of 679.82 ± 3.93 ng/g and 62.59 ± 3.32 ng/g, respectively. For short-chain PFHxA and PFBS, as well as the emerging PFAS GenX, the IAA-1 treatment showed higher concentrations compared with IAA-100. This trend is consistent with the observations for TG shoots at Day 54. Considering the similar PGR foliar application method, spray timing, and growth duration used in this study, the results suggest that IAA may enhance PFAS accumulation in plant tissues across different plant species. Overall, the tested IAA treatments demonstrated beneficial effects on PFAS accumulation under the experimental conditions examined. In Alfalfa roots, similar treatment trends were observed as well (**Figure S5**).

For the IBA treatments, IBA-10 resulted in higher PFAS concentrations in Alfalfa shoots, with values of 1567.33 ± 99.14 ng/g, 294.95 ± 24.77 ng/g, 2862.54 ± 211.78 ng/g, and 167.44 ± 6.83 ng/g for the long-chain compounds PFOA, PFNA, PFHxS, and PFOS, respectively, whereas IBA-100 showed higher concentrations for PFBS and GenX. These concentrations were approximately 2-5 times higher than those observed in the control.

Similar to PFAS, IBA application resulted in higher Cd accumulation (mg/kg) in Stellaria Media (Chickweed) grown in Cd-spiked soil (25 mg/kg) [12]. The dry shoot biomass of *S. media* was 1.114 ± 0.02 g, 1.154 ± 0.01 g, 1.170 ± 0.007 g, 1.238 ± 0.021 g, and 1.188 ± 0.018 g for the control (Cd only), IBA (25 mg/L), IBA (50 mg/L), IBA (75 mg/L), and IBA (100 mg/L) treatments, respectively. Although the biomass yield had no positive correlation with IBA dose, the Cd concentration in the shoots was increased from 45.66 ± 0.92 mg/kg (control) to, 47.57 ± 0.62

mg/kg (IBA, 25 mg/L) and 52.06 ± 2.36 mg/kg (IBA, 50 mg/L). With IBA at 75 and 100 mg/L, however, the Cd concentration decreased to 44.48 ± 3.10 mg/kg and 40.61 ± 0.85 mg/kg in the same order.

References

1. Fujii, S.; Polprasert, C.; Tanaka, S.; Hong Lien, N. P.; Qiu, Y., New POPs in the water environment: distribution, bioaccumulation and treatment of perfluorinated compounds–a review paper. *Journal of Water Supply: Research and Technology—AQUA* **2007,** *56* (5), 313-326.
2. Steinle-Darling, E.; Reinhard, M., Nanofiltration for trace organic contaminant removal: structure, solution, and membrane fouling effects on the rejection of perfluorochemicals. *Environmental science & technology* **2008,** *42* (14), 5292-5297.
3. Kim, S.; Chen, J.; Cheng, T.; Gindulyte, A.; He, J.; He, S.; Li, Q.; Shoemaker, B. A.; Thiessen, P. A.; Yu, B.; Zaslavsky, L.; Zhang, J.; Bolton, E. E., PubChem in 2021: new data content and improved web interfaces. *Nucleic Acids Research* **2021,** *49* (D1), D1388-D1395.
4. Zhou, Q.; Deng, S.; Yu, Q.; Zhang, Q.; Yu, G.; Huang, J.; He, H., Sorption of perfluorooctane sulfonate on organo-montmorillonites. *Chemosphere* **2010,** *78* (6), 688-694.
5. Christensen, E. R.; Wang, Y.; Huo, J.; Li, A., Properties and fate and transport of persistent and mobile polar organic water pollutants: A review. *Journal of Environmental Chemical Engineering* **2022**, 107201.
6. Brooke, D.; Footitt, A.; Nwaogu, T., Environmental risk evaluation report: Perfluorooctanesulphonate (PFOS). **2004**.
7. Pauletto, P. S.; Bandosz, T. J., Activated carbon versus metal-organic frameworks: A review of their PFAS adsorption performance. *Journal of Hazardous Materials* **2022,** *425*, 127810.
8. Liu, X.; Zhu, C.; Yin, J.; Li, J.; Zhang, Z.; Li, J.; Shui, F.; You, Z.; Shi, Z.; Li, B., Installation of synergistic binding sites onto porous organic polymers for efficient removal of perfluorooctanoic acid. *Nature Communications* **2022,** *13* (1), 2132.
9. Zhang, W.; Zhang, D.; Liang, Y., Nanotechnology in remediation of water contaminated by poly- and perfluoroalkyl substances: A review. *Environmental pollution* **2019,** *247*, 266-276.
10. Zhang, W.; Cao, H.; Mahadevan Subramanya, S.; Savage, P.; Liang, Y., Destruction of perfluoroalkyl acids accumulated in Typha latifolia through hydrothermal liquefaction. *ACS Sustainable Chemistry & Engineering* **2020,** *8* (25), 9257-9262.
11. Zhang, W.; Zhang, Q.; Liang, Y., Ineffectiveness of ultrasound at low frequency for treating per- and polyfluoroalkyl substances in sewage sludge. *Chemosphere* **2022,** *286*, 131748.
12. Lin, L.; Ma, Q.; Wang, J.; Lv, X.; Liao, M. a.; Xia, H.; Chen, S.; Lai, Y.; Chen, C.; Wang, X., Effects of indole‐3‐butytric acid (IBA) on growth and cadmium accumulation in the accumulator plant Stellaria media. *Environmental Progress & Sustainable Energy* **2018,** *37* (2), 733-737.
